# Anti-Melanogenic Effect of *Dendropanax morbiferus* and Its Active Components *via* Protein Kinase A/Cyclic Adenosine Monophosphate-Responsive Binding Protein- and p38 Mitogen-Activated Protein Kinase-Mediated Microphthalmia−Associated Transcription Factor Downregulation

**DOI:** 10.3389/fphar.2020.00507

**Published:** 2020-04-23

**Authors:** Jung Up Park, Seo Young Yang, Rui Hong Guo, Hong Xu Li, Young Ho Kim, Young Ran Kim

**Affiliations:** ^1^ College of Pharmacy and Research Institute of Drug Development, Chonnam National University, Gwangju, South Korea; ^2^ College of Pharmacy, Chungnam National University, Daejeon, South Korea

**Keywords:** *Dendropanax morbiferus*, (10E)-9,16-dihydroxyoctadeca-10,17-dien-12,14-diynoate, anti-melanogenesis, tyrosinase, PKA/CREB, p-p38/p38, microphthalmia−associated transcription factor

## Abstract

*Dendropanax morbiferus* H. Lév has been reported to have some pharmacologic activities and also interested in functional cosmetics. We found that the water extract of *D. morbiferus* leaves significantly inhibited tyrosinase activity and melanin formation in α-melanocyte stimulating hormone (MSH)-induced B16-F10 cells. *D. morbiferus* reduced melanogenesis-related protein levels, such as microphthalmia−associated transcription factor (MITF), TRP-1, and TRP-2, without any cytotoxicity. Two active ingredients of *D. morbiferus,* (10E)-9,16-dihydroxyoctadeca-10,17-dien-12,14-diynoate (DMW-1) and (10E)-(–)-10,17-octadecadiene-12,14-diyne-1,9,16-triol (DMW-2) were identified by testing the anti-melanogenic effects and then by liquid chromatography-tandem mass spectrometry (LC/MS/MS) analysis. DMW-1 and DMW-2 significantly inhibited melanogenesis by the suppression of protein kinase A (PKA)/cyclic AMP (cAMP)-responsive binding protein (CREB) and p38 MAPK phosphorylation. DMW-1 showed a better inhibitory effect than DMW-2 in α-MSH-induced B16-F10 cells. *D. morbiferus* and its active component DMW-1 inhibited melanogenesis through the downregulation of cAMP, p-PKA/CREB, p-p38, MITF, TRP-1, TRP-2, and tyrosinase. These results indicate that *D. morbiferus* and DMW-1 may be useful ingredients for cosmetics and therapeutic agents for skin hyperpigmentation disorders.

## Introduction

*Dendropanax morbiferus* H. Lév, an endemic species in South Korea, has been used as an alternative traditional medicine for several diseases, such as headache, dysmenorrhea, infectious disorders, and skin disorders, for a long time (Jeong et al., 1995; Park et al., 2004; Lim et al., 2015). There are some reports that *D. morbiferus* shows anti-hyperuricemic, anti-amnesic, anti-obesity, immunomodulatory, and anti-inflammatory activities (Birhanu et al., 2018; Cho et al., 2018; Park et al., 2018a; Park et al., 2018b; Song et al., 2018; Sun et al., 2018b; Choo et al., 2019). The corresponding ingredients isolated from *D. morbiferus* leaves, such as 1-tetradecanol, dendropanoxide, rutin, gentisic acid, or oleifoliosides, have been reported to have diverse therapeutic potentials (Yu et al., 2012; Lee et al., 2013; Park et al., 2014; Park et al., 2017; Sun et al., 2018a). Recently, *D. morbiferus* is increasingly interested in functional materials in cosmetics. Several studies have reported that *D. morbiferus* and its components have diverse therapeutic potentials, such as anti-wrinkle, hair growth, and moisturizing effect (Lee et al., 2005; Deng et al., 2010; Lee et al., 2015; Park and Han, 2016). In addition, 1-tetradecanol, and β-sitosterol isolated from *D. morbiferus* were reported to possess anti-melanogenic effects (Lee et al., 2015). However, there are few studies about the anti-melanogenic activity of *D. morbiferus* and its components as a therapeutic potential herbal medicine. To further investigate the efficacy and mechanism of *D. morbiferus*, we evaluated its function on the anti-melanogenic effect in this study.

Melanin is produced from melanocytes in the epidermis by a pigmentation process called melanogenesis and it is the main factor determining the color of human skin, hair, and eyes (Riley, 2003; D'Orazio et al., 2013). In addition, melanin plays an important role in protecting the skin from ultraviolet (UV) radiation (Costin and Hearing, 2007; Brenner and Hearing, 2008). Moreover, abnormal accumulation of melanin on skin surface leads to various skin pigmentation disorders, such as dots, freckles, and post-inflammatory hyperpigmentation (Pillaiyar et al., 2018). Melanogenesis regulation is under a complex control. Tyrosinase, the main melanogenic factor in the biosynthesis of melanin, is a rate-limiting enzyme that catalyzes the hydroxylation of L-tyrosine to form tyrosine to 3,4-dihydroxyphenylalanine (DOPA) and oxidizes DOPA to produce DOPA-quinone (Slominski et al., 1988; Slominski et al., 2012). The main role of the melanin-synthesizing tyrosinase gene family (tyrosinase, TRP-1, and TRP-2) is to differentiate, proliferate, and accumulate melanin into melanocytes. Microphthalmia−associated transcription factor (MITF) is not only the main regulator of melanocyte proliferation, development, and survival but also a major regulator of melanogenesis-related protein expression (Liu and Fisher, 2010; Kim et al., 2018b). cAMP-responsive binding protein (CREB) regulates the expression of MITF in melanosomes. The phosphorylation of CREB is regulated by the activation of cAMP/protein kinase A (PKA) cascades that are known to play main roles in melanin synthesis (Pillaiyar et al., 2017). The mitogen-activated protein kinase (MAPK) family proteins, including extracellular signal-regulated kinase (ERK) 1/2, c-Jun N terminal kinase (JNK) 1/2 and p38, are known to play crucial roles in melanogenesis (Wu et al., 2011; Pillaiyar et al., 2017). Melanin production can be triggered by a variety of factors, including α-melanocyte stimulating hormone (α-MSH), adrenocorticotropic hormone (ACTH), and stem cell factor (SCF) (Chan et al., 2014). Specifically, α-MSH is a key regulator in the biosynthesis of melanin and prompt expression of cAMP.

Here, we found that *D. morbiferus* inhibited tyrosinase activity and melanin formation in melanocytes. In the present study, we have investigated the molecular mechanisms of *D. morbiferus* on anti-melanogenic effects by testing MITF, TRP-1, TRP-2, cAMP, PKA/CREB, and p38 MAPK pathways. In addition, the active ingredients from *D. morbiferus* were identified by LC/MS/MS analysis.

## Material and Methods

### Chemical and Reagents

L-3,4-dihydroxyphenylalanine (L-DOPA), α-melanocyte stimulating hormone (α-MSH), and arbutin were purchased from Sigma-Aldrich Chemical Co. (St. Louis, MO, USA). Dulbecco's modified Eagle's medium (DMEM) was obtained from Welgene (DG, KR). Fetal bovine serum (FBS) and penicillin/streptomycin were purchased from Thermo Fisher Scientific (MA, USA). Lysis buffer and 3-(4, 5-dimethylthiazol-2-yl)-5-(3 carboxymethoxyphenyl)-2-(4-sulfophenyl)-2H-tetrazolium (MTS) were purchased from Promega (WI, USA). Antibodies specific to MITF, tyrosinase, TRP-1, TRP-2, p38, and GAPDH were purchased from Santa Cruz Biotechnology (CA, USA). Antibodies specific to PKA, CREB, ERK, JNK, p-CREB, p-PKA, p-p38, p-ERK, and p-JNK proteins were obtained from Cell Signaling Technology (MA, USA). Horseradish peroxidase-conjugated secondary antibodies were purchased from Jackson ImmunoResearch, Inc. (PA, USA). Western Bright™ ECL reagent was purchased from Advansta, Inc. (CA, USA). All other chemicals were used in analytical reagent grade.

### Preparation of the Water Extract From *D. morbiferus* Leaves and Its Fractions

*D. morbiferus* water extract (W-DP) and its ethyl acetate fraction (W-EA) were prepared using a method described previously (Park et al., 2018a). *D. morbiferus* leaves were obtained from Jangheung (JL, KR) and the origin of the herbal medicine was confirmed by Jeollanam-do institute of natural resources research (JL, KR). *D. morbiferus* leaves were extracted once with water at 100°C for 4 h. The filtered extract was concentrated using a continuous vacuum evaporator (40°C, 670 mmHg) followed by lyophilization in a vacuum drier (770 mmHg). The crude water extract of *D. morbiferus* leaves (W-DP, 20 g) was suspended in water and successively divided with 3 x 1 L volumes of n-hexane, chloroform, ethyl acetate, n-butanol, and aqueous fraction, respectively. The fractions were concentrated using a rotary vacuum concentrator and dried in a 50°C dry oven for >48 h as described previously (Park et al., 2018a).

### Cell Cultures and Cell Viability Assay

Murine B16-F10 skin melanoma cells supplied by the Korea Cell Line Bank (SEL, KR) were maintained in DMEM supplemented with 1% penicillin/streptomycin and 10% FBS under an atmosphere of 5% CO_2_ in a humidified 37°C incubator. To test cell viability, B16-F10 cells were cultured into 96-well plates (SPL Life Sciences Co., GG, KR) at a density of 0.5 x 10^4^ cells/well overnight. The cells were treated with W-DP at different concentrations with or without α-MSH (200 nM) for an additional 72 h. The viability of B16-F10 cells was determined by MTS according to the manufacturer's instructions, and absorbance was read at 490 nm with an ELx808 ELISA microplate reader (BioTek Instruments, Inc., VT, USA).

### Intracellular Tyrosinase Activity

Intracellular tyrosinase activity was tested according to previously described method (Li et al., 2018). B16-F10 cells (1 x 10^5^ cells/well) were treated with W-DP (0.03, 0.1, 0.3, 0.5 mg/ml), W-EA (0.01, 0.03 mg/ml), or arbutin (0.54 mg/ml) for 2 h, followed by addition with α-MSH (200 nM) for 72 h. The cells were then lysed in a lysis buffer with protease inhibitor cocktail for 30 min at 4°C and centrifuged at 13,000 rpm for 10 min at 4°C. Proteins were quantified by using Bradford reagent and equal amounts of the lysates were treated with L-DOPA (2 mg/ml) in 96-well plates at 37°C for 2 h. The production of DOPAchrome was measured using an ELISA microplate reader at an absorbance of 490 nm.

### Melanin Content Assay

B16-F10 cells (1 x 10^5^ cells/well) were incubated into six-well plate (SPL Life Sciences Co., GG, KR) at 37°C overnight. B16-F10 cells were pretreated with W-DP (0.03, 0.1, 0.3 mg/ml), W-EA (0.01 mg/ml), or arbutin (0.54 mg/ml) for 2 h prior to stimulation with α-MSH (200 nM) for 72 h. The cells were dissolved in 1 N NaOH with 10% DMSO for 1 h at 60°C. Total melanin lysates were transferred to a 96-well plate and the optical absorbance was measured at 405 nm using an ELISA microplate reader.

### Preparation of Active Compounds From *D. morbiferus*

Dried leaves and stems of *Dendropanax morbiferus* H. Lév were purchased from the herbal company Hanna Arboretum, Goheung, Jeollanam-do, Korea and taxonomically identified by one of the authors (Prof. YHK). A voucher specimen (CNU 17002) was deposited at the Herbarium of the College of Pharmacy, Chungnam National University. *D. morbiferus* (3.0 kg) were extracted with 10-fold volume of boiling methanol (10 L × 3) under reflux condition for three times. The methanol extract (470.0 g) was suspended in water and partitioned with *n*-hexane, CH_2_Cl_2_, EtOAc, and *n*-BuOH to yield *n*-hexane fraction, CH_2_Cl_2_ fraction, EtOAc fraction, *n*-BuOH fraction, and aqueous fraction, respectively.

Column chromatography (CC) separations were performed using silica gels (SiO_2_; 70–230, 230–400 µm particle size; Fuji Silysia Chemical Ltd., AIC, JP) and C_18_ resins (75 µm, Fuji Silysia Chemical Ltd., AIC, JP). Thin-layer chromatography (TLC) separations were performed using pre-coated silica gels 60 F254 and reversed-phase F254S plates (Merck, Darmstadt, Germany). The NMR spectra were recorded using a JEOL ECA 600 spectrometer (1H, 600 MHz; 13C, 150 MHz; JEOL Ltd, TKY, JP). High-resolution electrospray ionization mass spectra (HRESIMS) were obtained using an Agilent 6530 accurate-mass quadrupole-time of flight-liquid chromatography/mass spectrometry (Q-TOF LC/MS).

The EtOAc extract (21.8 g) was subjected to a silica gel column chromatography with a gradient of CHCl_3_–MeOH (60:1–0:1) to give ten fractions (Fr. EA1–EA10). Fraction EA3 (900.0 mg) was chromatographed on a silica gel column using a gradient of n-hexane–EtOAc (6:1–3:1) elution solvent to give eight subfractions (EA3A–EA3H), then subfraction EA3C (70.0 mg) was chromatographed on a reverse–phase column with a gradient of MeOH–water (9:1–1:0) to obtain three subfractions (EA3C1–EA3C3), subfraction EA3C1 (10.0 mg) was further chromatographed on a reverse–phase column with MeOH–water (3:1) to yield DMW-1 (5.3 mg). Subfraction EA3D (238.0 mg) was chromatographed on a reverse–phase column with a gradient of MeOH–water (3:7–1:0) to give eleven subfractions (EA3D1–EA3D11), then subfraction EA3D9 (13.6 mg) was further purified by a silica gel column chromatography with *n*-hexane–acetone (1:1) to yield DMW-2 (6.2 mg). (10E)-9,16-dihydroxyoctadeca-10,17-dien-12,14-diynoate (DMW-1): C_19_H_26_O_4_; viscous liquid; [α] –38.0° (c = 0.1, MeOH); HRESIMS ([M+NH4]+ m/z 336.2167, calcd for 336.2175); 1D-NMR (CD3OD, 600 MHz), and 2D-NMR data (CD3OD, 600 MHz) (see **Supplementary Figure S2** and **Table S1**). (10E)-(–)-10,17-Octadecadiene-12,14-diyne-1,9,16-triol (DMW-2): C_18_H_26_O_3_; viscous liquid; [α] –32.6° (c = 0.1, MeOH); HRESIMS ([M+NH4]+ m/z: 308.2219, calcd for 308.2226); 1D-NMR (CD3OD, 600 MHz), and 2D-NMR data (CD3OD, 600 MHz) (see **Supplementary Figure S3** and **Table S1**). Stock solutions of samples were diluted using phosphate buffered saline (PBS) or ethanol and filter-sterilized and then diluted with PBS for the working concentrations.

### Liquid Chromatography-Tandem Mass Spectrometry (LC/MS/MS) Analysis

Methanol (Sigma-Aldrich Chemical Co., MO, USA) and formic acid (Kanto, Chemical, TKY, JP) were used as LC/MS/MS gradient solvents. A Shimadzu HPLC system (KT, JP) consisted of a dual solvent pump LC-20A, an autosampler SIL-20AC, and a column oven Gemini C_18_. In addition, a mass spectrometer Q-Trap 4000 (AB Sciex, Foster City, CA) was used for the separation and detection of the single compounds. In detail, the separation was carried out on a Gemini C_18_ column (CA, USA) (5 µm, 50 mm× 2.0 mm). The column was heated to 40°C under a flow rate of 0.3 ml/min with a stepwise elution from mobile phase A (0.1% formic acid in water) to mobile phase B (0.1% formic acid in acetonitrile) as follows. The linear gradient elution was set at 15–60% B for 1–4 min, 60% B for 5 min, and 15% B for 10–12 min. The injection volume was set at 10 μl. The mass spectrometer was operated in positive Turbo Ion Spray mode and multiple reaction monitoring (MRM) scan mode. The instrumental mass parameters were set as follows: ion spray voltage 5500 V, curtain gas 20, GS1 60, GS2 60, and desolvation temperature (TEM) 500°C. First, we identified the full scan of mass spectra and product ion mass spectra of DMW-1 and 2. The mass transition of m/z 301.212 to m/z 55.2 was used for DMW-1, and m/z 273.213 to m/z 55.1 was used for DMW-2. AB Sciex Analyst software (version 1.5.2) was used for data integration.

The stock solutions of DMW-1 and 2 were prepared at a concentration of 100 µM in methanol. The linear range of the calibration curve for DMW-1 and 2 was diluted in water at concentrations of 5, 20, 50, 200, and 500 ng/ml and used as a working standard. The stock solution of W-DP was prepared in water at concentrations of 10 and 50 mg/ml. The solutions were stored at 4°C.

### Cyclic AMP Assay

B16-F10 cells were cultured at a density of 5 x 10^4^ cells/well in 24-well plates for 24 h and pretreated with W-DP (0.1 mg/ml), W-EA (0.01 mg/ml), DMW-1 (10 µM), or arbutin (0.54 mg/ml) for 2 h, followed by treatment with α-MSH (200 nM) for 24 h. The cells were incubated with cell lysis buffer at −80°C overnight, and then, the thawed lysates were harvested and centrifuged at 10,000 rpm for 10 min at 4°C. The cAMP levels in the lysates were measured by ELISA following the manufacturer's experimental protocols (R&D Systems, MN, USA).

### Western Blot Analysis

The target proteins were detected by Western blot analysis with specific antibodies. B16-F10 cells were cultured in 6-well plates at a density of 1.5 x 10^5^ cells/well and then incubated overnight. The cells were pretreated with W-DP (0.1 mg/ml), W-EA (0.01 mg/ml), DMW-1 (10 µM), DMW-2 (10 µM), or arbutin (0.54 mg/ml) for 2 h before stimulation with α-MSH (200 nM) for 24 h. Then, cell lysates were harvested and centrifuged at 13,000 rpm for 10 min at 4°C. Equal amounts of the lysates were electrophoresed on 10% sodium dodecyl sulfate-polyacrylamide gel electrophoresis (SDS-PAGE) and transferred to polyvinylidene difluoride membranes (PVDF) (Millipore, MA, USA) at 400 mA for 2 h. The membranes were incubated with appropriate primary antibodies against MITF, tyrosinase, TRP-1, TRP-2, PKA, CREB, ERK, JNK, p-38, p-PKA, p-CREB, p-ERK, p-JNK, p-p38, or GAPDH at 4°C overnight. After being washed, the membranes were incubated with horseradish peroxidase-conjugated anti-rabbit or anti-mouse immunoglobulin secondary antibodies for 1 h. Immunoreactive proteins were conducted using Western Bright™ ECL reagents and a C300 chemiluminescence imager (Azure Biosystems, Inc., CA, USA).

### Statistical Analysis

All experiments were repeated in at least three separate experiments. Statistical differences were evaluated using GraphPad Prism version 5.01 (San Diego, CA, USA). The results shown are representative experiments. We used one-way ANOVA for multi-group comparisons followed by a Tukey *post hoc* test; *p* < 0.05 was considered statistically significant.

## Results

### Effect of W-DP on Cell Viability, Tyrosinase Activity and Melanin Formation in α-MSH-Induced B16-F10 Cells

**Figure 1:**
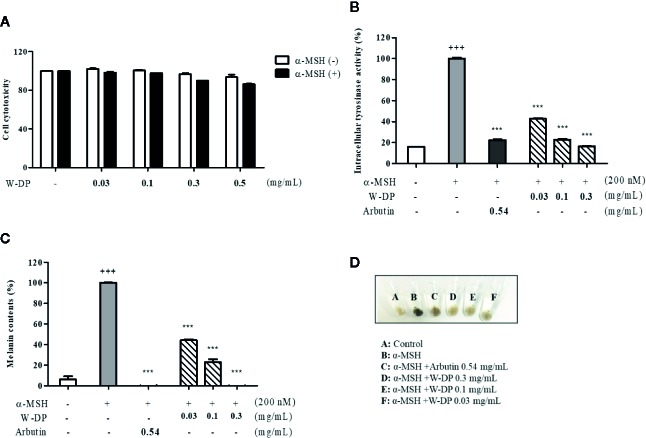
Effect of W-DP on melanogenesis in α-MSH-induced B16-F10 cells. B16-F10 cells pretreated with different concentrations of W-DP (0.03, 0.1, 0.3, 0.5 mg/ml) were incubated with or without α-MSH (200 nM) for 72 h. **(A)** Cell viability was tested by MTS. **(B)** The cells were then lysed and quantified by using Bradford reagent. Equal amounts of the lysates were treated with L-DOPA (2 mg/ml) in 96-well plate at 37°C for 2 h. The production of DOPAchrome was measured using an ELISA microplate reader at an absorbance of 490 nm. **(C)** The cells were dissolved in 1 N NaOH with 10% DMSO for 1 h at 60°C. Total melanin lysates were transferred to a 96-well plate and the optical absorbance was measured at 405 nm using an ELISA microplate reader. **(D)** Colors of the pellets indicated the change of melanogenesis from α-MSH-induced B16-F10 cells. Data are expressed as the mean ± SEM of three independent experiments. ^***^ *P* < 0.001 versus α-MSH stimulation. ^+++^ *P* < 0.001 versus the untreated control.

We studied the effect of W-DP on melanogenesis in B16-F10 melanoma cells. First, the cell viability was tested after treatment with various concentrations of W-DP in the presence or absence of α-MSH. W-DP showed no cytotoxicity to B16-F10 cells (**Figure 1A**). Next, W-DP (0.03, 0.1, 0.3, 0.5 mg/ml) significantly reduced intracellular tyrosinase activity and melanin production in a dose-dependent manner (**Figures 1B, C**). In addition, the W-DP remarkably decreased the melanin contents in the cell pellets, which showed lighter colors (**Figure 1D**). These results suggest that W-DP decreases melanin synthesis through the downregulation of tyrosinase.

### Effect of W-DP on the Expression of Melanogenesis-Related Proteins in α-MSH-Induced B16-F10 Cells

**Figure 2:**
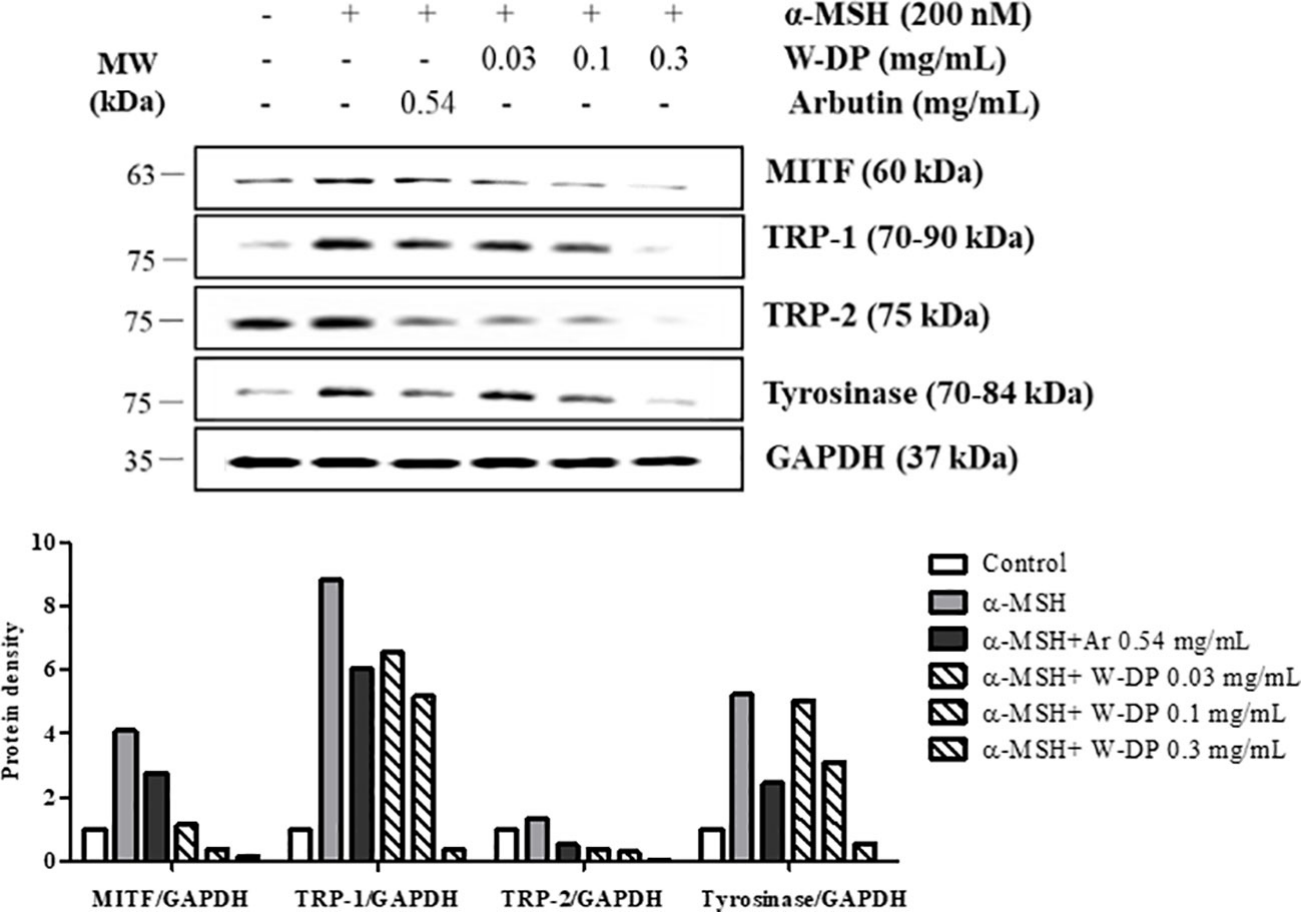
Effect of *Dendropanax morbiferus* water extract (W-DP) on the expression of melanogenesis-related proteins in α-MSH-induced B16-F10 cells. B16-F10 cells were pretreated with various concentrations of W-DP (0.03, 0.1, 0.3 mg/ml) or arbutin (0.54 mg/ml) for 2 h and then stimulated with α-MSH (200 nM) for 24 h. The protein levels of MITF, tyrosinase, TRP-1, and TRP-2 were detected by Western blot analysis. Arbutin was used as a positive control, and GAPDH was used as the loading control. MITF, tyrosinase, TRP-1, and TRP-2 were analyzed and quantified using Azure Spot software.

We tested the inhibitory effect of W-DP on melanogenesis-related proteins by Western blot analysis. The expression levels of MITF and tyrosinase were significantly reduced by treatment with W-DP in a dose-dependent manner. TRP-1 and TRP-2 stimulated by α-MSH were also simultaneously decreased by W-DP treatment (**Figure 2**). Our results suggest that its effect on melanin synthesis may be associated with the suppression of melanogenesis-related proteins such as tyrosinase, TRP-1, and TRP-2 through MITF inhibition.

### Structure Elucidation of DMW-1 and 2 in *D. morbiferus*

**Figure 3:**
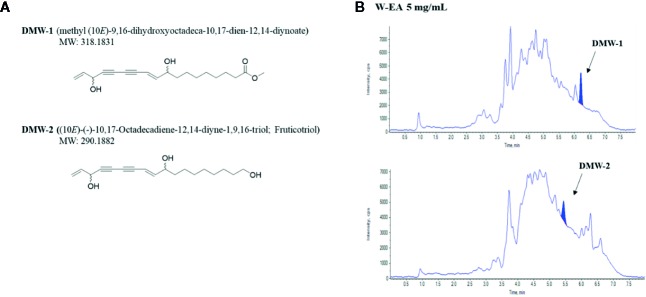
Chemical structures and chromatograms of DMW-1 and 2. **(A)** Chemical structures of DMW-1 and 2 and **(B)** total ion chromatogram of DMW-1 and 2 in W-EA at 5 mg/ml. W-EA, ethyl acetate fraction.

To identify the anti-melanogenic active constituents, we isolated 22 components from the ethyl acetate fraction (W-EA), which was separated from *D. morbiferus* by continuous fractionation (see **Supplementary Figure S1**). The crude water extract of *D. morbiferus* leaves was subjected to a succession of fractionation procedures and the W-EA showed the most significant inhibitory effects. We evaluated the effects of W-EA at various concentrations in α-MSH-activated B16-F10 cells. W-EA significantly inhibited tyrosinase activity in a dose-dependent manner. W-EA at the concentrations of ~0.001, 0.01, and 0.03 mg/ml showed 45%, 53%, and 65% inhibitory effects, respectively. W-EA was confirmed to be sufficiently effective even at a concentration of 0.01 mg/ml lower than 0.03 mg/ml. We showed the one concentration of W-EA results to compare the efficacy of single ingredients (data not shown). Of all isolated components, (10*E*)-9,16-dihydroxyoctadeca-10,17-dien-12,14-diynoate (DMW-1) and (10*E*)-(–)-10,17-octadecadiene-12,14-diyne-1,9,16-triol (DMW-2) significantly suppressed melanin contents in α-MSH-induced B16-F10 cells (**Supplementary Table S2**). DMW-1 had a molecular formula of C_19_H_26_O_4_ by HRESIMS ([M+NH_4_]^+^ *m*/*z* 336.2167, calcd for 336.2175) and obtained as a viscous liquid (see **Supplementary Figure S2**). The 1H-NMR spectrum showed signals for five olefinic protons at *δ* 6.34 (dd, *J* = 15.9, 5.7 Hz), 5.94 (ddd, *J* = 17.1, 10.2, 5.4 Hz), 5.79 (ddd, *J* = 15.9, 1.6, 0.9 Hz), 5.42 (d, *J* = 17.1 Hz), 5.22 (d, *J* = 10.2 Hz) and 4.96 (dd, *J* = 5.4, 1.1 Hz), two oxygenated methane protons at *δ* 4.96 (dd, *J* = 5.4, 1.1 Hz) and 4.13 (dtd, *J* = 7.2, 5.7, 1.6 Hz), and one methoxy proton at *δ* 3.76 (s). The ^13^C-NMR spectrum revealed the presence of a carbonyl carbon at *δ* 176.0, four olefinic carbons at *δ* 151.9, 138.1, 116.6, and 108.4, four quaternary carbons at *δ* 82.2, 78.0, 74.0, and 70.6, two oxygen-bearing carbons at *δ* 72.4 and 64.0, and one methoxy carbon at *δ* 52.0 (see **Supplementary Figure S2** and **Table S1**). The above analyses suggested that DMW-1 should be a polyacetylene compound. The NMR results of DMW-1 were similar to those of the known DMW-2, except for a methoxy-carbonyl group (see **Supplementary Figure S3** and **Table S1**). DMW-1 and DMW-2 have a negative optical rotation value ([α] –38.0° and –32.6°), indicating the same relative configuration. The structures of DMW-1 and 2 components were identified by NMR spectroscopy and are shown in **Figure 3A**. DMW-1 and 2 were quantified in W-EA by LC/MS/MS analysis. The DMW-2 content (637 ng/ml) was dramatically higher than the DMW-1 content (87.1 ng/ml) in the W-EA fraction (**Figure 3B**). The reaction times of DMW-1 and DMW-2 were 6.2 and 5.43 min, respectively.

### Inhibitory Effect of DMW-1 and 2 on Melanogenesis in α-MSH-Induced B16-F10 Cells

**Figure 4:**
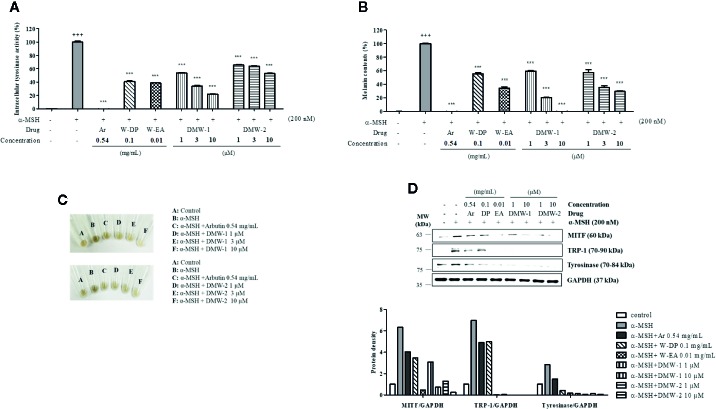
Effects of DMW-1 and 2 on melanogenesis in α-MSH-induced B16-F10 cells. B16-F10 cells (1 x 10^5^ cells/well) were incubated into six-well plate at 37°C overnight. B16-F10 cells were pretreated with W-DP (0.1 mg/ml), W-EA (0.01 mg/ml), DMW-1 (1, 3, 10 µM), DMW-2 (1, 3, 10 µM), or arbutin (0.54 mg/ml) for 2 h prior to stimulation with α-MSH (200 nM) for 72 h. **(A)** Equal amounts of the lysates were treated with L-DOPA (2 mg/ml) at 37°C for 2 h. The production of DOPAchrome was measured at an absorbance of 490 nm. **(B)** The cells were dissolved in 1 N NaOH with 10% DMSO for 1 h at 60°C. Total melanin lysates were measured at an absorbance of 405 nm. **(C)** Colors of the pellets indicated the change of melanogenesis from α-MSH-induced B16-F10 cells. **(D)** The expression levels of MITF, tyrosinase, and TRP-1 were determined by Western blot analysis, and the protein bands were quantified using Azure Spot software. GAPDH was used as the loading control. Data are expressed as the mean ± SEM of three independent experiments. ^***^P < 0.001 versus α-MSH stimulation. ^+++^ *P* < 0.001 versus the untreated control. Ar, arbutin; W-DP, *D. morbiferus* water extract; W-EA, ethyl acetate fraction.

To examine the effects of DMW-1 and 2 on melanogenesis, B16-F10 cells were pretreated with two compounds and then stimulated with α-MSH for 72 h. DMW-1 and 2 showed significant decreases in tyrosinase activity and melanin production. Notably, DMW-1 showed a better inhibitory effect than DMW-2 in B16-F10 cells. DMW-1 reduced the intracellular tyrosinase activity with an estimated IC_50_ of 1.175 μM and decreased the melanin accumulation with an estimated IC_50_ of 1.221 μM. The inhibitory effects of DMW-1 approached the effect of the reference compound arbutin (**Figures 4A, B**). As shown in **Figure 4C**, both compounds resulted in decreased melanin contents with lighter colors. Based on the above results, we studied whether DMW-1 and 2 could inhibit melanogenesis-related protein expression by Western blot analysis. DMW-1 and 2 remarkably decreased the protein expression levels of MITF, tyrosinase, and TRP-1 (**Figure 4D**). These results indicate that the anti-melanogenic effects of DMW-1 and 2 may be mediated by downregulating melanogenesis-related proteins.

### Effect of DMW-1 and 2 on the Phosphorylation Levels of PKA and CREB in α-MSH-Induced B16-F10 Cells

**Figure 5:**
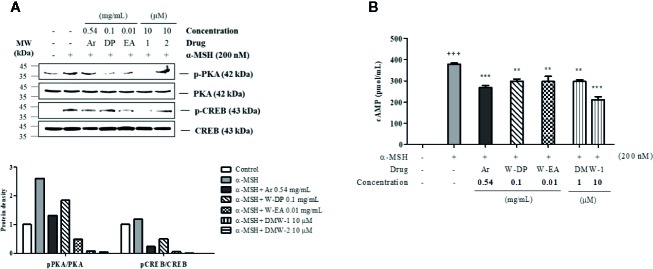
Effects of DMW-1 and 2 on the phosphorylation levels of PKA and CREB in α-MSH-induced B16-F10 cells. The cells were pretreated with W-DP (0.1 mg/ml), W-EA (0.01 mg/ml), DMW-1 (10 µM), DMW-2 (10 µM), or arbutin (0.54 mg/ml) for 2 h before stimulation with α-MSH (200 nM) for 24 h. **(A)** The p-PKA and p-CREB protein levels were determined by Western blot analysis, and normalized to total PKA and CREB. The phosphorylation levels of PKA and CREB were analyzed and quantified using Azure Spot software. **(B)** The lysates were collected and cAMP levels were tested by ELISA. Data are expressed as the mean ± SEM of three independent experiments. ^**^P < 0.01, and ^***^P < 0.001 versus α-MSH stimulation. ^+++^ *P* < 0.001 versus the untreated control. Ar, arbutin; W-DP, *D. morbiferus* water extract; W-EA, ethyl acetate fraction.

To investigate the effect of DMW-1 and 2 on cAMP-related signaling pathway, phosphorylation levels of PKA and CREB were analyzed by Western blot analysis. Compared to W-DP and W-EA, the phosphorylation levels of PKA and CREB proteins were markedly reduced by the treatment of DMW-1 and 2 in α-MSH-induced B16-F10 cells. However, PKA and CREB levels remained unchanged. DMW-1 showed a slightly greater inhibitory effect on the phosphorylation levels of PKA/CREB than DMW-2 in B16-F10 cells (**Figure 5A**). Moreover, the cAMP level accumulated in the lysates was evaluated using competitive ELISA. As shown in **Figure 5B**, DMW-1 decreased intracellular cAMP levels in a dose-dependent manner.

### Effect of DMW-1 and 2 on the Phosphorylation of p38 in α-MSH-Stimulated B16-F10 Cells

**Figure 6:**
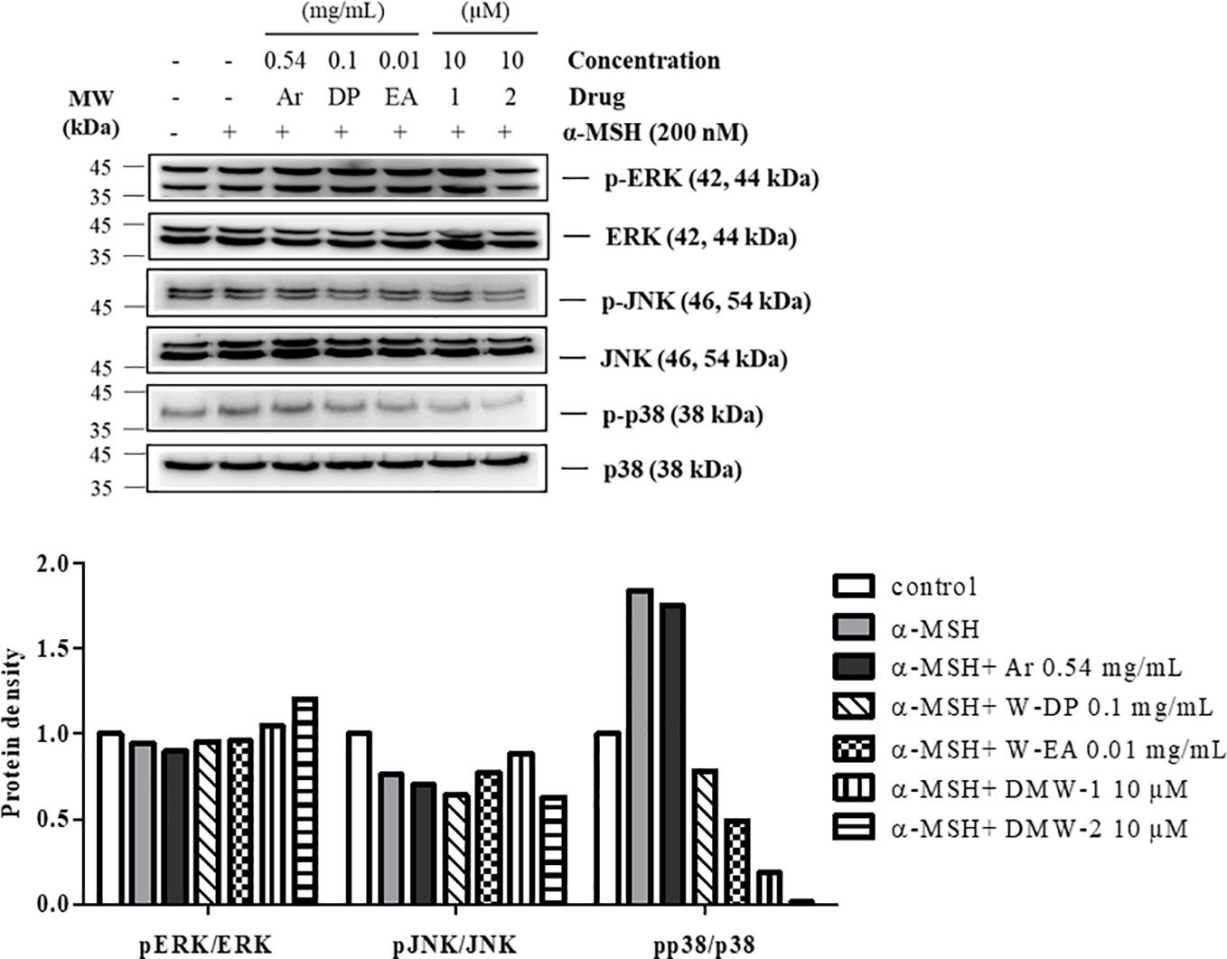
Effect of DMW-1 and 2 on p38 phosphorylation in α-MSH-stimulated B16-F10 cells. B16-F10 cells were pretreated with W-DP (0.1 mg/ml), W-EA (0.01 mg/ml), DMW-1 (10 µM), DMW-2 (10 µM), or arbutin (0.54 mg/ml) for 2 h before stimulation with α-MSH (200 nM) for 24 h. Phosphorylation levels of ERK, JNK, and p38 were determined by Western blot analysis. Equal levels of proteins were confirmed using total ERK, JNK, and p38 antibodies. The protein bands were analyzed and quantified using Azure Spot software. Ar, arbutin; W-DP, *D. morbiferus* water extract; W-EA, ethyl acetate fraction.

To further investigate whether DMW-1 and 2 exert anti-melanogenic effect through regulating the MAPK pathway, the phosphorylation levels of MAPKs were assayed by Western blot analysis. As shown in **Figure 6**, DMW-1 and 2 significantly decreased p38 phosphorylation. In contrast, the phosphorylation levels of ERK and JNK proteins showed no increase after treatment with DMW-1 and 2 in B16-F10 cells. These data indicate that the anti-melanogenic activity of DMW-1 is associated with the decrease of p-p38 as well as p-CREB.

## Discussion

There is an increasing interest in medicinal plants and their bioactive compounds for novel whitening therapies in recent years. Various cosmetic ingredients such as arbutin, kojic acid, and vitamin C have been used to inhibit hyperpigmentation (Ng et al., 2014). We have studied to identify herbal medicines showing anti-melanogenic effects without cytotoxicity. To the best of our knowledge, this is the first report to demonstrate the anti-melanogenic effect of *D. morbiferus* leaves and its new single constituents. In this study, we investigated the underlying mechanisms of *D. morbiferus* -mediated anti-melanogenic effects and identified the active compounds. Interestingly, *D. morbiferus* significantly decreased melanin content in α-MSH-induced B16-F10 cells (**Figure 1**). *D. morbiferus* did not show any cytotoxicity to B16-F10 cells (**Figure 1A**). Melanogenesis regulation is under complex control. Melanin synthesis can be regulated by the direct suppression of tyrosinase and by the downregulation of tyrosinase or other melanogenesis-related proteins. Here, we have investigated the molecular mechanisms of *D. morbiferus* on anti-melanogenic effects focusing on tyrosinase, TRP-1, TRP-2, MITF, cAMP, PKA/CREB, and p38 MAPK pathways. Interestingly, *D. morbiferus* markedly reduced the expression levels of MITF, TRP-1, TRP-2, and tyrosinase in a dose-dependent manner (**Figure 2**). Next, we isolated 22 components from ethyl acetate fraction of *D. morbiferus*. Of all the isolated components, DMW-1 and 2 significantly inhibited melanin contents in α-MSH-induced B16-F10 cells (**Supplementary Table S2**). DMW-1 and 2 are polyacetylene-based compounds with similar structures (**Figure 3**), and their structural explanations are provided in the supplementary data (**Supplementary Figures S2** and **S3** and **Table S1**). Of all polyacetylene-based compounds, several studies have reported that (9Z,16S)-16-hydroxy-9,17-octadecadiene-12,14-diynoic acid has anti-obesity, anti-osteoclastogenic, antioxidant, anti-inflammatory, and anticomplement activities (Park et al., 2004; Kang et al., 2018; Kim et al., 2018a). The beneficial effect of (2Z, 8Z)-matricaria acid methyl ester has been reported to inhibit tyrosinase activity (Luo et al., 2009). In this study, we confirmed that DMW-1 and 2 dramatically suppressed tyrosinase activity and melanin overproduction. Consistently, the expression levels of MITF, tyrosinase, and TRP-1 were remarkably decreased by DMW-1 and 2 (**Figure 4**). We found that DMW-1 and 2 dramatically inhibited melanogenesis through the downregulation of MITF and tyrosinase. DMW-1 showed significant inhibitory effect on melanogenesis, which indicated that the active compound was abundant in *D. morbiferus*. CREB regulates the expression of MITF in melanosomes and the phosphorylation of CREB is regulated by the activation of cAMP/PKA cascades that are known to play main roles in melanin synthesis (Pillaiyar et al., 2017). The inhibitory effect of DMW-1 on the phosphorylation levels of PKA and CREB was slightly greater than DMW-2 in B16-F10 cells, and DMW-1 significantly decreased intracellular cAMP levels in a dose-dependent manner (**Figure 5**). Next, we examined the role of the MAPK signaling pathway in the effects of two chemical constituents on melanogenesis. DMW-1 and 2 significantly decreased p38 phosphorylation in B16-F10 cells. In contrast, the phosphorylation levels of ERK and JNK were not increased by the treatment of DMW-1 and 2 in B16-F10 cells (**Figure 6**). These results suggested that the PKA/CREB and p38-mediated signaling pathways were involved in melanin production and tyrosinase activity, which were affected by DMW-1. These results indicate that DMW-1 isolated from *D. morbiferus* has anti-melanogenic effect *via* PKA/CREB- and p38 MAPK-mediated MITF degradation.

**Figure 7:**
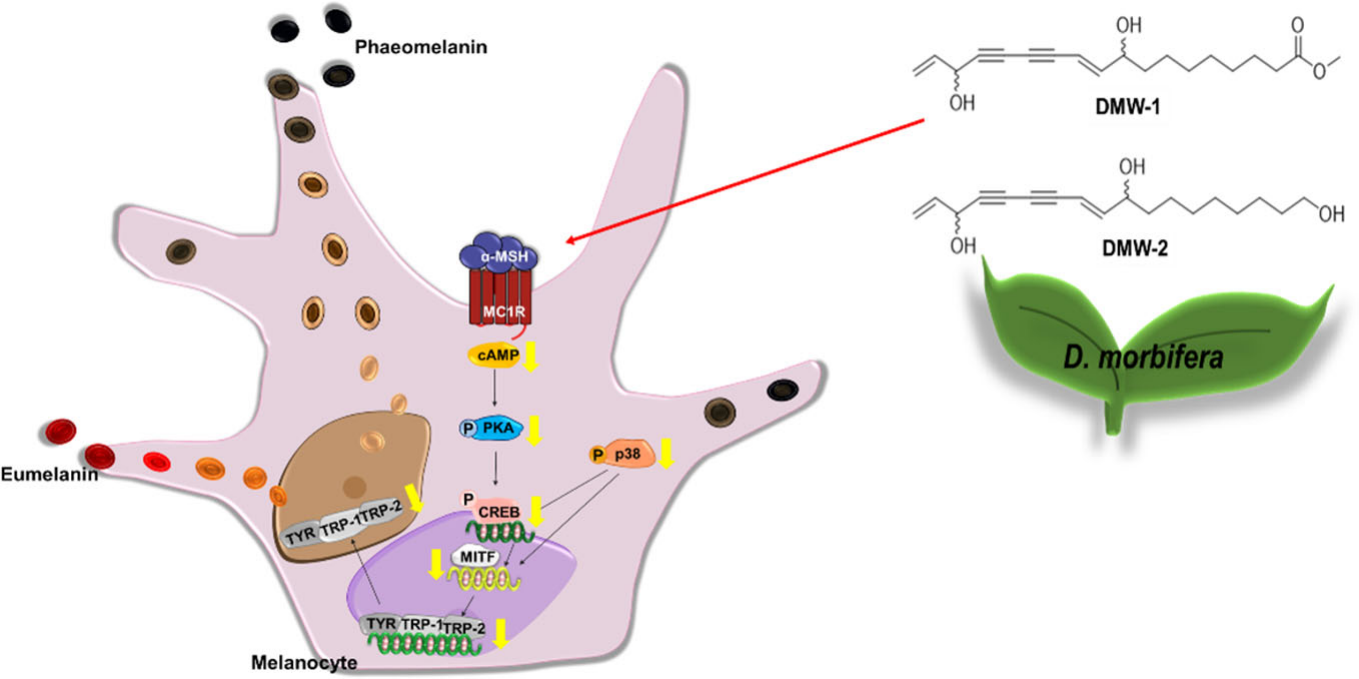
Underlying potential mechanism of anti-melanogenesis of *D. morbiferus.* The anti-melanogenesis effect of *D. morbiferus* might be associated with downregulation of MITF. *D. morbiferus* and its active component DMW-1 significantly inhibited melanin formation by inhibition of melanogenesis-related proteins such as tyrosinase, TRP-1, and TRP-2. *D. morbiferus* and DMW-1 show multifunctional inhibitory activities on melanogenesis pathways *via* PKA/CREB- and p38 MAPK-mediated MITF degradation.

In conclusion, *D. morbiferus* and its active component DMW-1 significantly inhibited melanin formation by inhibition of melanogenesis-related proteins such as tyrosinase, TRP-1, and TRP-2, without any cytotoxicity. *D. morbiferus* and DMW-1 show multifunctional inhibitory activities on melanogenesis pathways *via* PKA/CREB- and p38 MAPK-mediated MITF degradation (**Figure 7**). These results suggested that *D. morbiferus* and DMW-1 might be potential ingredients for application in cosmetics and therapeutic agents for skin hyperpigmentation disorders.

## Data Availability Statement

The datasets generated for this study are available on request to the corresponding authors.

## Author Contributions

JP was involved in the project design, carried out major experiments, and drafted the manuscript. SY and HL participated to extract the active compounds from *D. morbiferus* and reviewed the protocol. RG wrote and revised the manuscript. YRK and YHK conceived the project, provided reagents, and conceptual design, and wrote the manuscript. All authors read and approved the manuscript finally. 

## Conflict of Interest

The authors declare that the research was conducted in the absence of any commercial or financial relationships that could be construed as a potential conflict of interest.

## Abbreviations

ACTH, adrenocorticotropic hormone; cAMP, cyclic adenosine monophosphate; CC, column chromatography; CREB, cAMP-responsive binding protein; DOPA, 3,4-dihydroxyphenylalanine; ERK, extracellular signal-regulated kinase 1/2; JNK, c-Jun N terminal kinase 1/2; MAPK, mitogen-activated protein kinase; MC1R, melanocortin type 1 receptor; MITF, microphthalmia−associated transcription factor; PKA, protein kinase A; SCF, stem cell factor; TRP-1, tyrosinase-related protein-1; TRP-2, tyrosinase-related protein-2; UV, ultraviolet; α-MSH, α-melanocyte stimulating hormone.
