# Supplementary material for: Anti-Melanogenic Effect of Dendropanax morbiferus and Its Active Components via Protein Kinase A/Cyclic Adenosine Monophosphate-Responsive Binding Protein- and p38 Mitogen-Activated Protein Kinase-Mediated Microphthalmia−Associated Transcription Factor Downregulation
Source: Front Pharmacol. 2020 Apr 23;11:507. doi: 10.3389/fphar.2020.00507 (PMC7191003; doi:10.3389/fphar.2020.00507)
Supplement: Supplementary file 1 [file DataSheet_1.docx]

**Supplementary material**

**Figure S1**

**
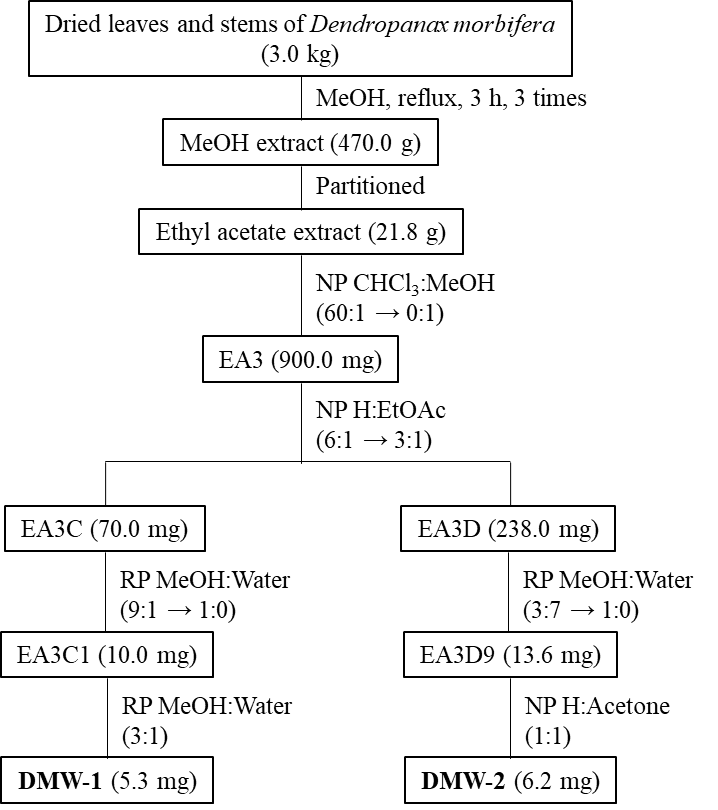
**

**Supplementary Figure S1.** Isolation scheme of compounds from *D. morbifera.* NP, normal phase column chromatography; RP, reverse phase column chromatography; MeOH, methanol; H, *n*-hexane; EtOAc, ethyl acetate; CHCl_3,_ chloroform.

**Figure S2**

**(A)**

**(B)**


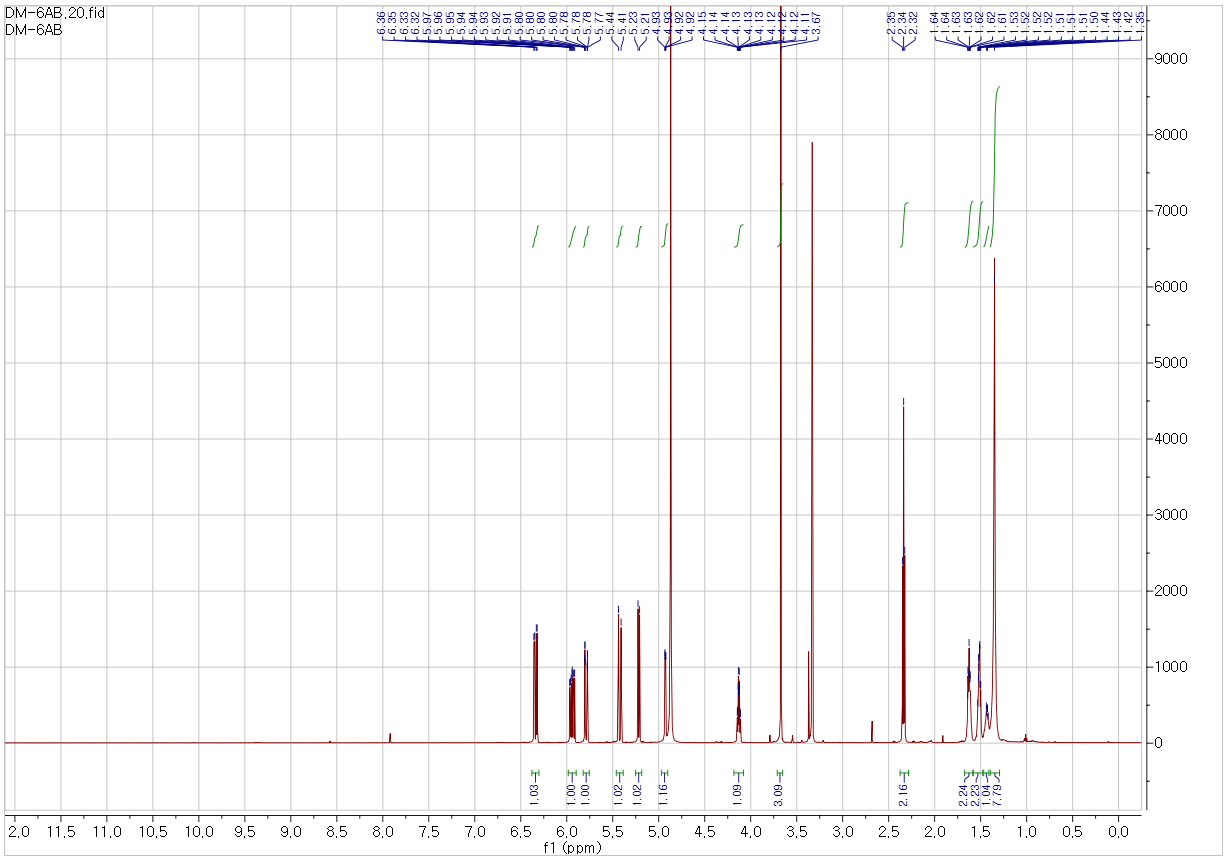


**(C)**


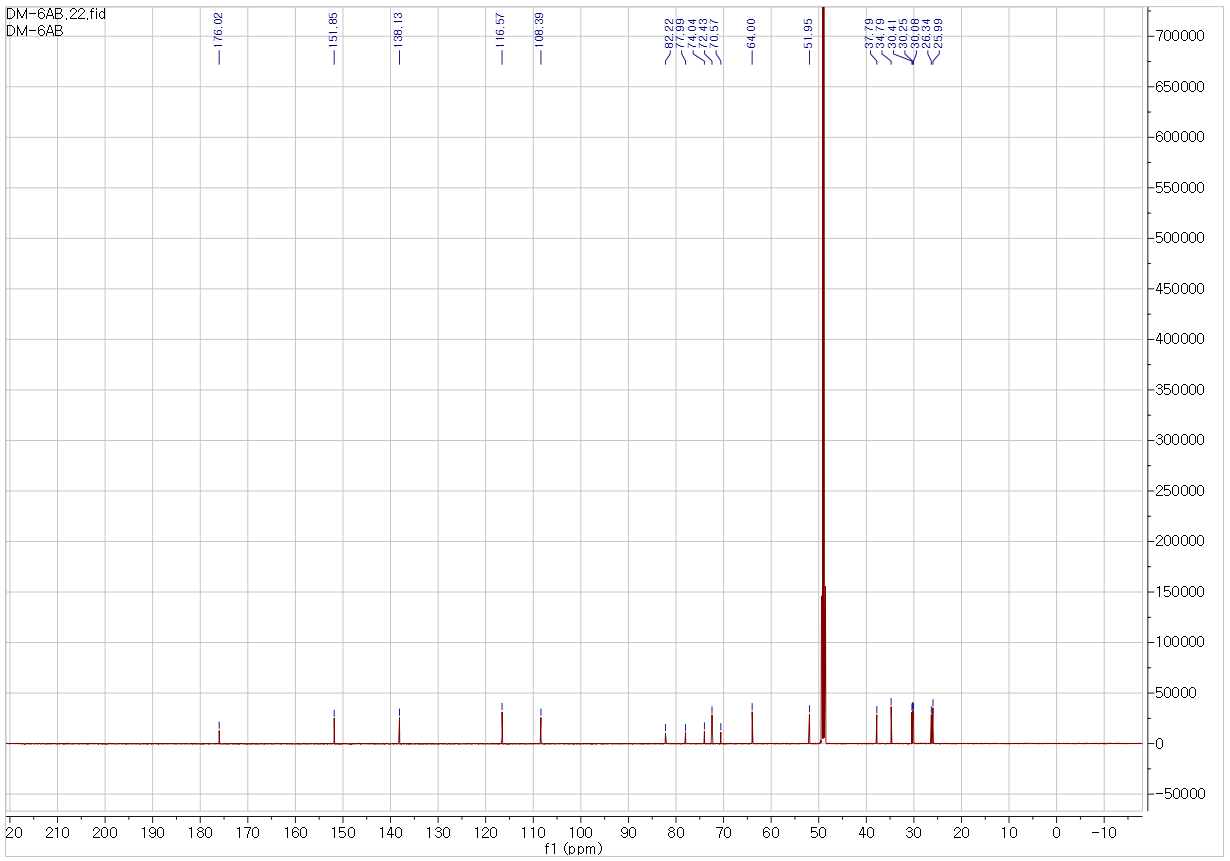


**(D)**


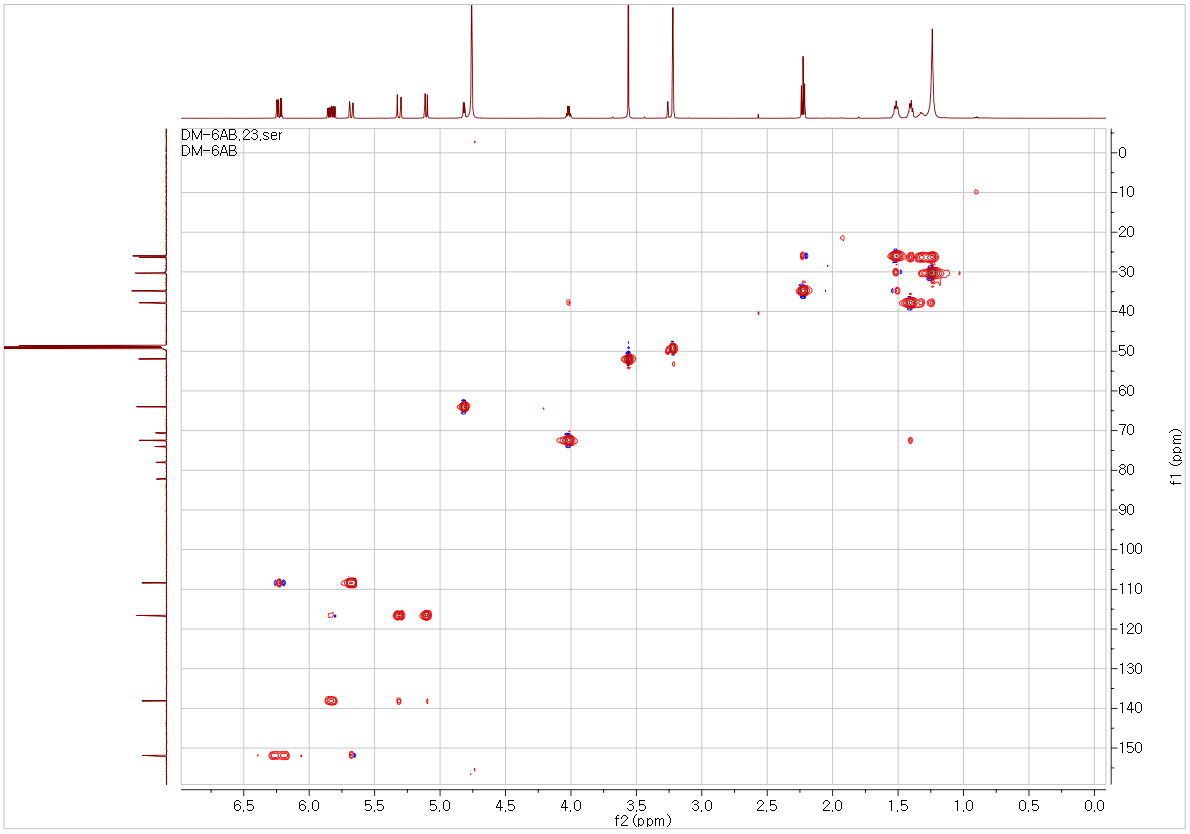


**(E)**


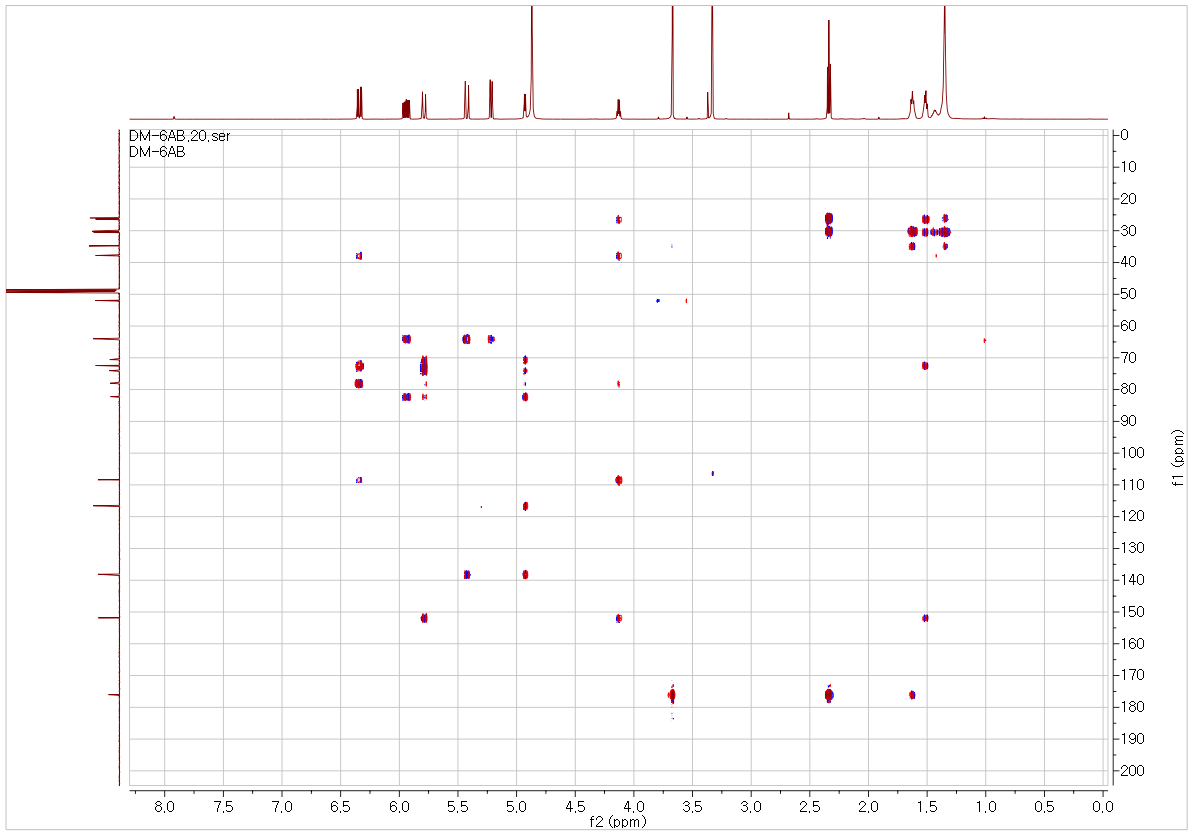


**(F)**


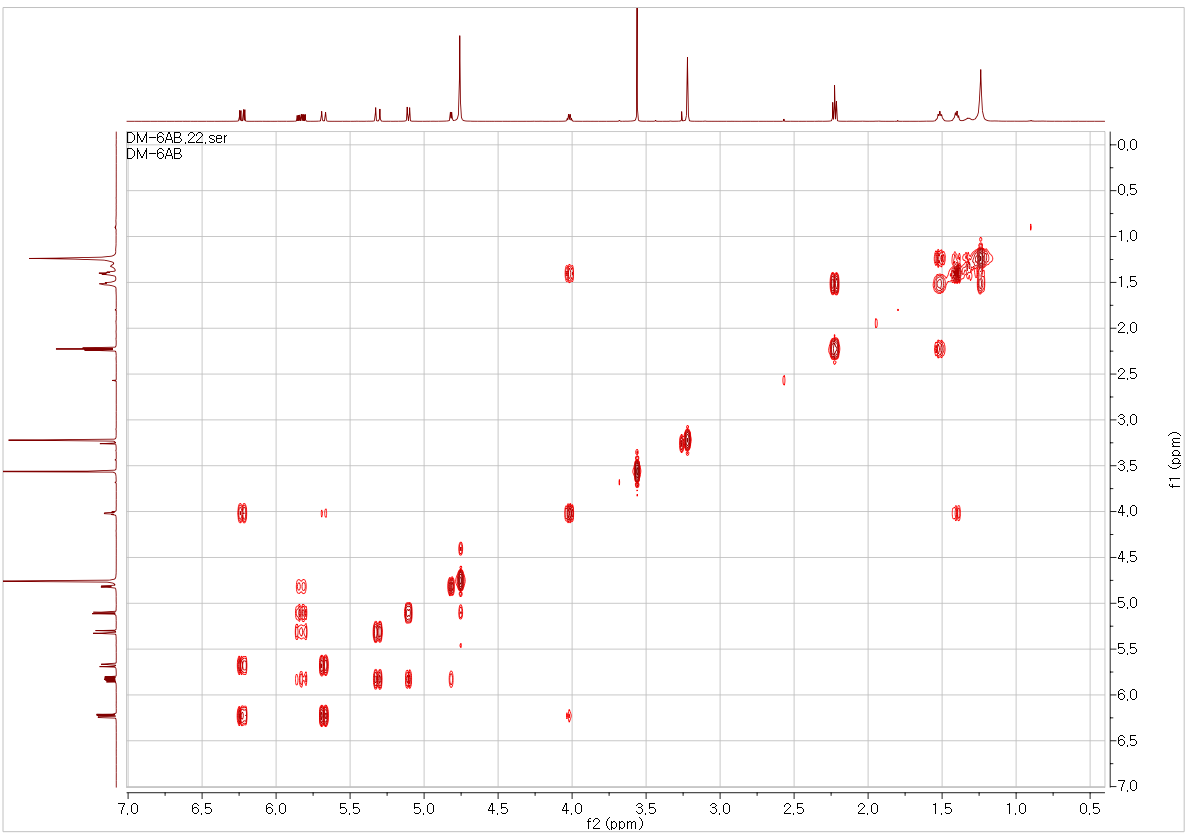


**(G)**

**Supplementary Figure S2.**Structure elucidation of DMW-1. C_19_H_26_O_4_, viscous liquid; [α] –38.0° (c = 0.1, MeOH); HRESIMS *m*/*z*: 336.2167 [M+NH_4_]^+^; 1D-NMR (CD_3_OD, 600 MHz) and 2D-NMR data (CD_3_OD, 600 MHz). (A) HMBC and COSY correlations of DMW-1, (B) ^1^H-NMR spectrum of DMW-1 in CD_3_OD (600 MHz), (C) ^13^C-NMR spectrum of DMW-1 in CD_3_OD (150 MHz), (D) HSQC spectrum of DMW-1 in CD_3_OD, (E) HMBC spectrum of DMW-1 in CD_3_OD, (F) COSY spectrum of DMW-1 in CD_3_OD, and (G) HRESIMS spectrum of DMW-1.

**Figure S3**

**(A)**

**(B)**


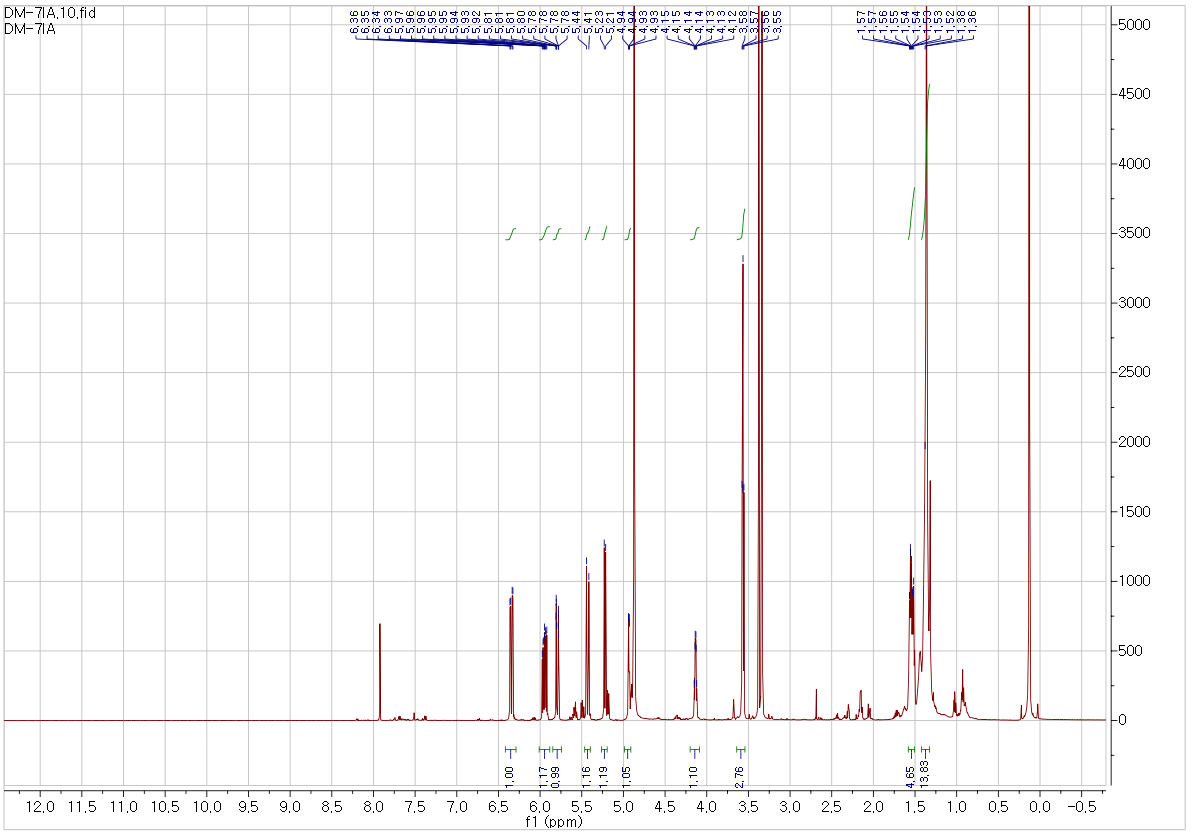


**(C)**


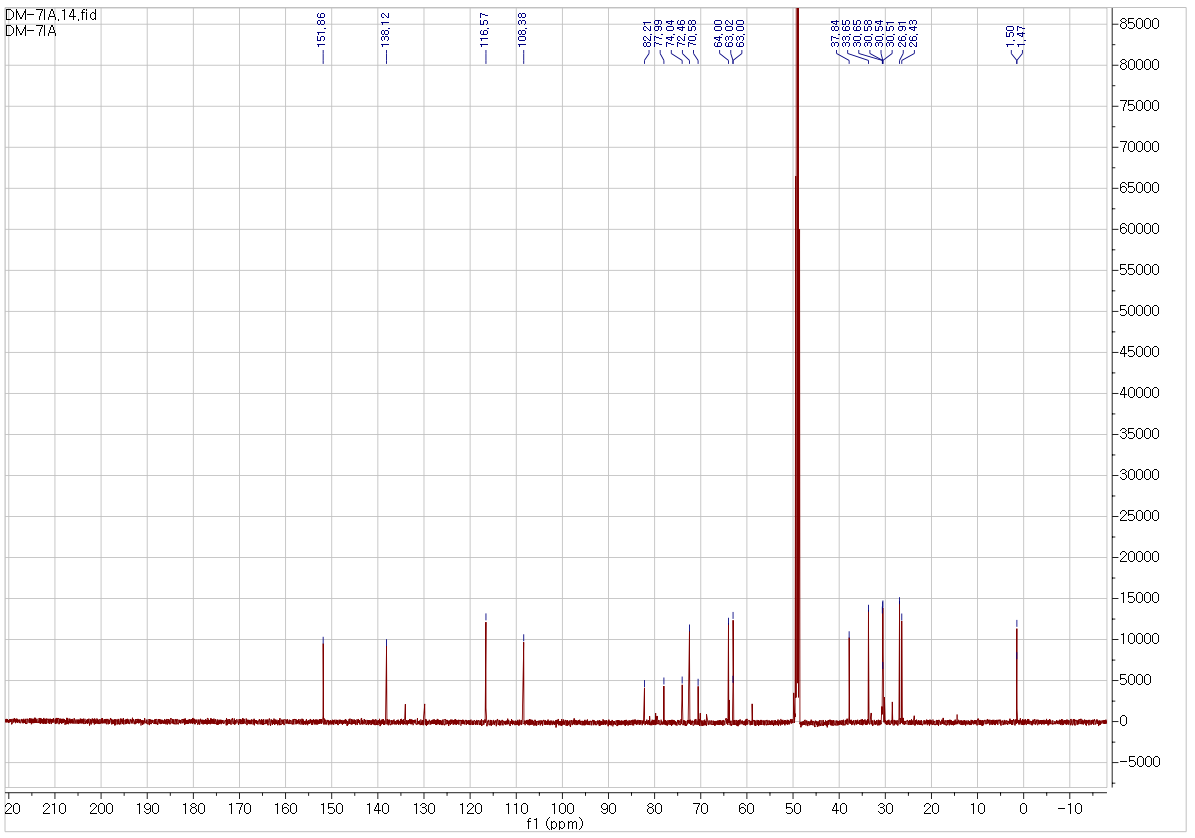


**(D)**


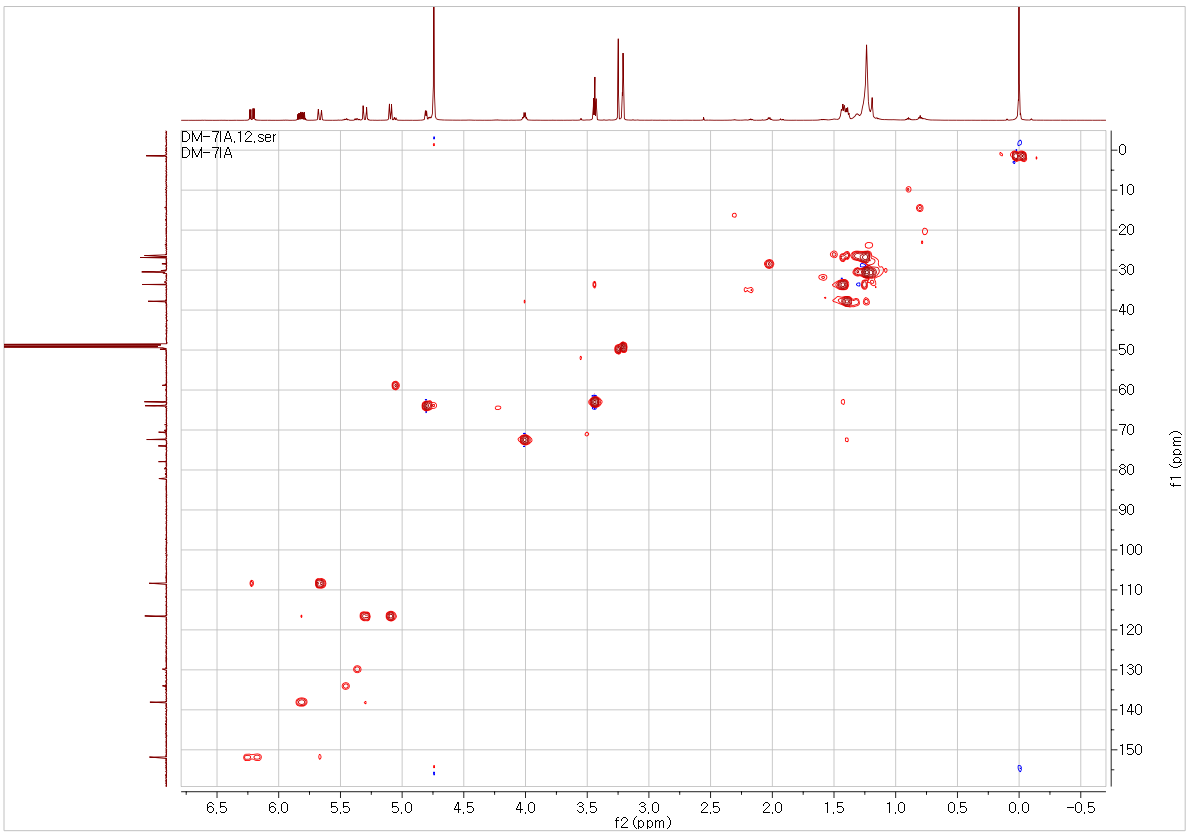


**(E)**


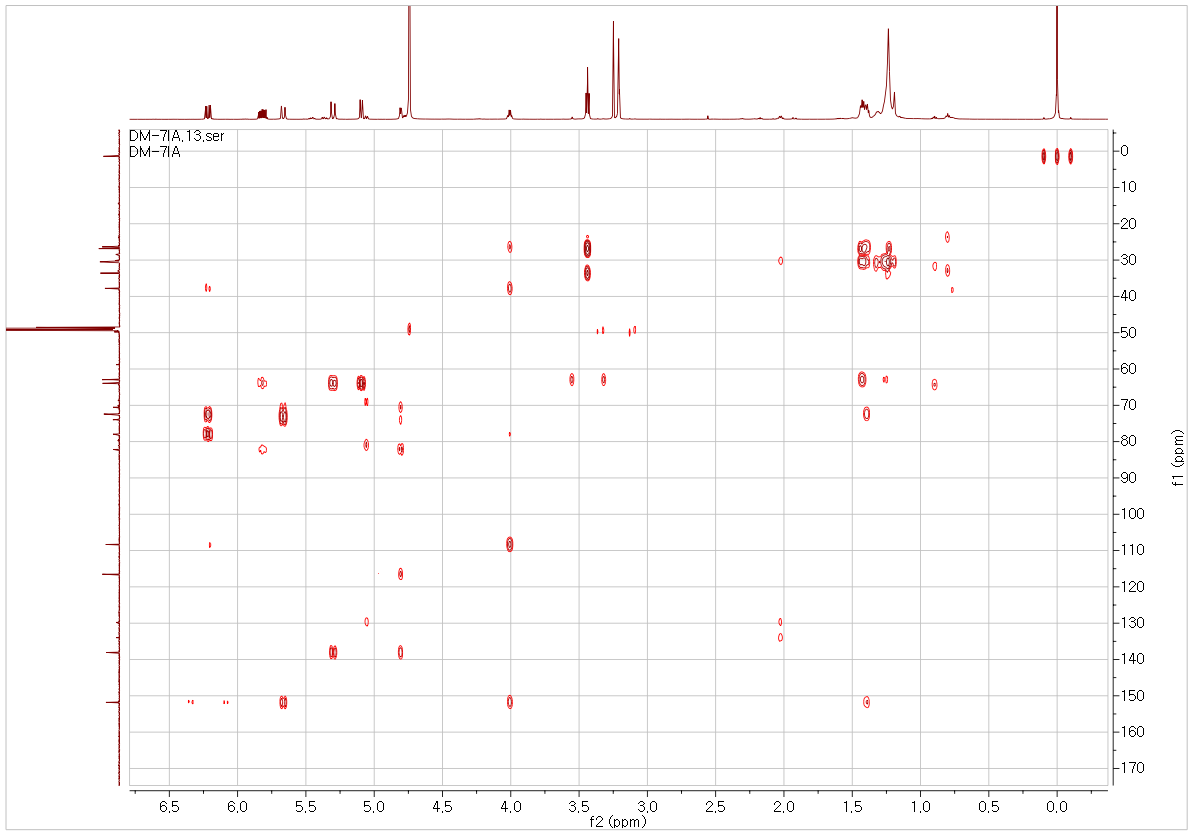


**(F)**


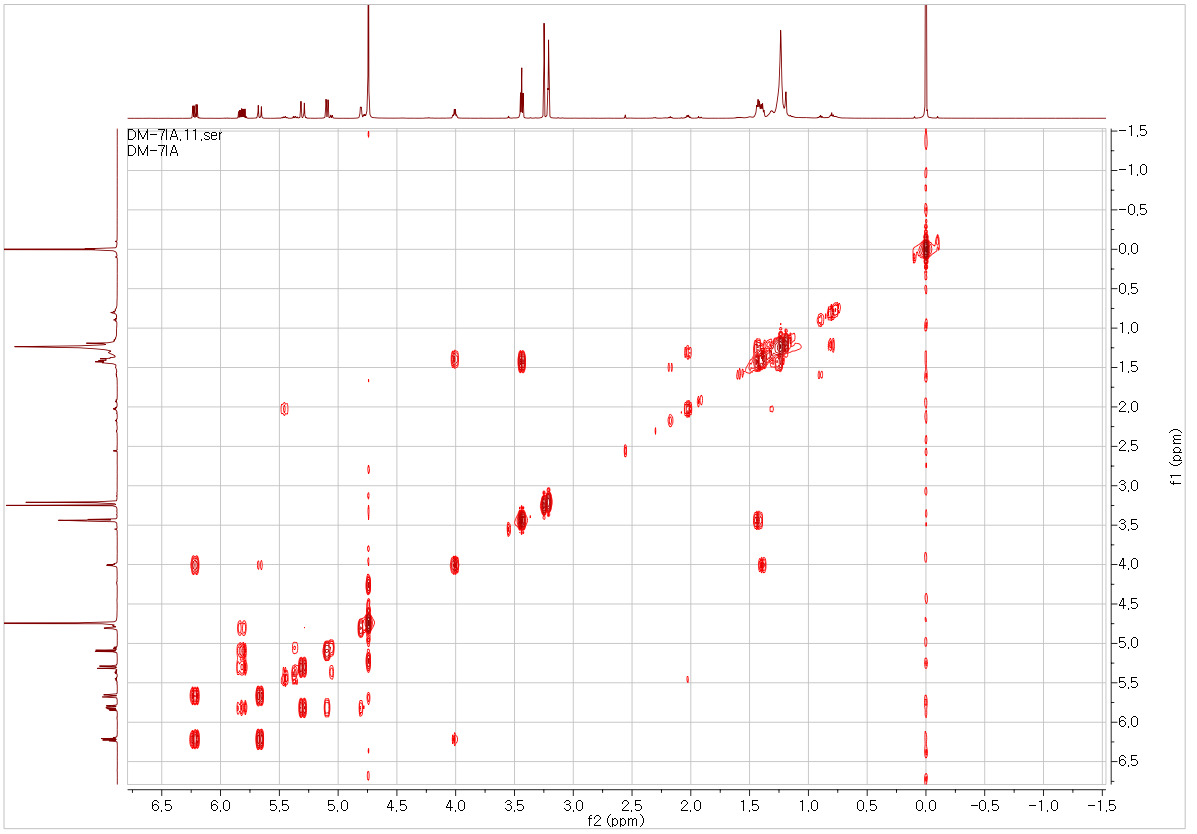


**(G)**

**Supplementary Figure S3.** Structure elucidation of DMW-2. C_18_H_26_O_3_, viscous liquid; [α] –32.6° (c = 0.1, MeOH); HRESIMS *m*/*z*: 308.2219 [M+NH_4_]^+^; 1D-NMR (CD_3_OD, 600 MHz) and 2D-NMR data (CD_3_OD, 600 MHz). (A) HMBC and COSY correlations of DMW-2, (B) ^1^H-NMR spectrum of DMW-2 in CD_3_OD (600 MHz), (C) ^13^C-NMR spectrum of DMW-2 in CD_3_OD (150 MHz), (D) HSQC spectrum of DMW-2 in CD_3_OD, (E) HMBC spectrum of DMW-2 in CD_3_OD, (F) COSY spectrum of DMW-2 in CD_3_OD, and (G) HRESIMS spectrum of DMW-2.

**Supplementary Table S1.** The NMR spectroscopic data of DMW-1 and 2

|  | **DMW-1** | | **DMW-2** | |
| --- | --- | --- | --- | --- |
| **Position** | δ_C_ | δ_H_ (mult., J in Hz) | δ_C_ | δ_H_ (mult., J in Hz) |
| **1** | 176.0 | - | 63.0 | 3.56, t (6.7) |
| **2** | 34.8 | 2.34, t (7.5) | 33.7 | 1.59*, m |
| **3** | 26.0 | 1.62, m | 26.9 | 1.37*, m |
| **4** | 30.4 | 1.35*, m | 30.7 | 1.37*, m |
| **5** | 30.3 | 1.35*, m | 30.6 | 1.37*, m |
| **6** | 30.1 | 1.35*, m | 30.5 | 1.37*, m |
| **7** | 26.3 | 1.43*, m | 26.4 | 1.54*, m |
| **8** | 37.8 | 1.51, dt (11.7, 3.6) | 37.8 | 1.53*, m |
| **9** | 72.4 | 4.13, dtd (7.2, 5.7, 1.6) | 72.5 | 4.14, m |
| **10** | 151.9 | 6.34, dd (15.9, 5.7) | 151.9 | 6.34, dd (15.9, 5.6) |
| **11** | 108.4 | 5.79, ddd (15.9, 1.6, 0.9) | 108.4 | 5.79, ddd (15.9, 1.6, 0.8) |
| **12** | 78.0 | - | 78.0 | - |
| **13** | 74.0 | - | 74.0 | - |
| **14** | 70.6 | - | 70.6 | - |
| **15** | 82.2 | - | 82.2 | - |
| **16** | 64.0 | 4.96, dd (5.4, 1.1) | 64.0 | 4.93, dd (5.5, 1.1) |
| **17** | 138.1 | 5.94, ddd (17.1, 10.2, 5.4) | 138.1 | 5.95, ddd (17.0, 10.2, 5.5) |
| **18** | 116.6 | 5.42, d (17.1)  5.22, d (10.2) | 116.6 | 5.43, d (17.1)  5.22, d (10.2) |
| **OCH_3_** | 52.0 | 3.67, s | - | - |

*Overlapped signals, assignments were done by HSQC, HMBC and COSY experiments.

**Supplementary Table S2.** Effect of single compounds on melanin contents in α-MSH-activated B16-F10 cells.

| **Compounds** | **Melanin contents (%)** | **Compounds** | | **Melanin contents (%)** | |
| --- | --- | --- | --- | --- | --- |
| **α-MSH** | 100 ± 2.05 | **Arbutin** | 85.96 ± 0.00 | |  |
| **1** | 84.06 ± 0.15 | **12** | 81.58 ± 0.00 | |  |
| **2** | 76.32 ± 1.46 | **13** | 83.33 ± 0.00 | |  |
| **3** | 83.19 ± 0.15 | **14** | 83.92 ± 0.00 | |  |
| **4** | 80.99 ± 0.00 | **15** | 80.26 ± 0.73 | |  |
| **5** | 77.34 ± 0.44 | **16** | 95.91 ± 0.29 | |  |
| **6** | 80.41 ± 0.88 | **17** | 80.99 ± 0.00 | |  |
| **7** | 97.37 ± 0.00 | **18** | 93.27 ± 0.00 | |  |
| **8** | 91.08 ± 0.15 | **19** | 83.63 ± 0.58 | |  |
| **9** | 84.06 ± 0.15 | **20** | 93.57 ± 0.29 | |  |
| **10** | 83.19 ± 0.73 | **21 (DMW-1)** | 62.28 ± 0.00 | |  |
| **11** | 85.38 ± 0.58 | **22 (DMW-2)** | 71.93 ± 0.00 | |  |

*Concentrations; α-MSH, 200 nM; Arbutin, 0.54 mg/mL; single compounds, 10 µM
