# Supplementary figures and images for: Anti-Melanogenic Effect of Dendropanax morbiferus and Its Active Components via Protein Kinase A/Cyclic Adenosine Monophosphate-Responsive Binding Protein- and p38 Mitogen-Activated Protein Kinase-Mediated Microphthalmia−Associated Transcription Factor Downregulation
Source: Front Pharmacol. 2020 Apr 23;11:507. doi: 10.3389/fphar.2020.00507 (PMC7191003; doi:10.3389/fphar.2020.00507)

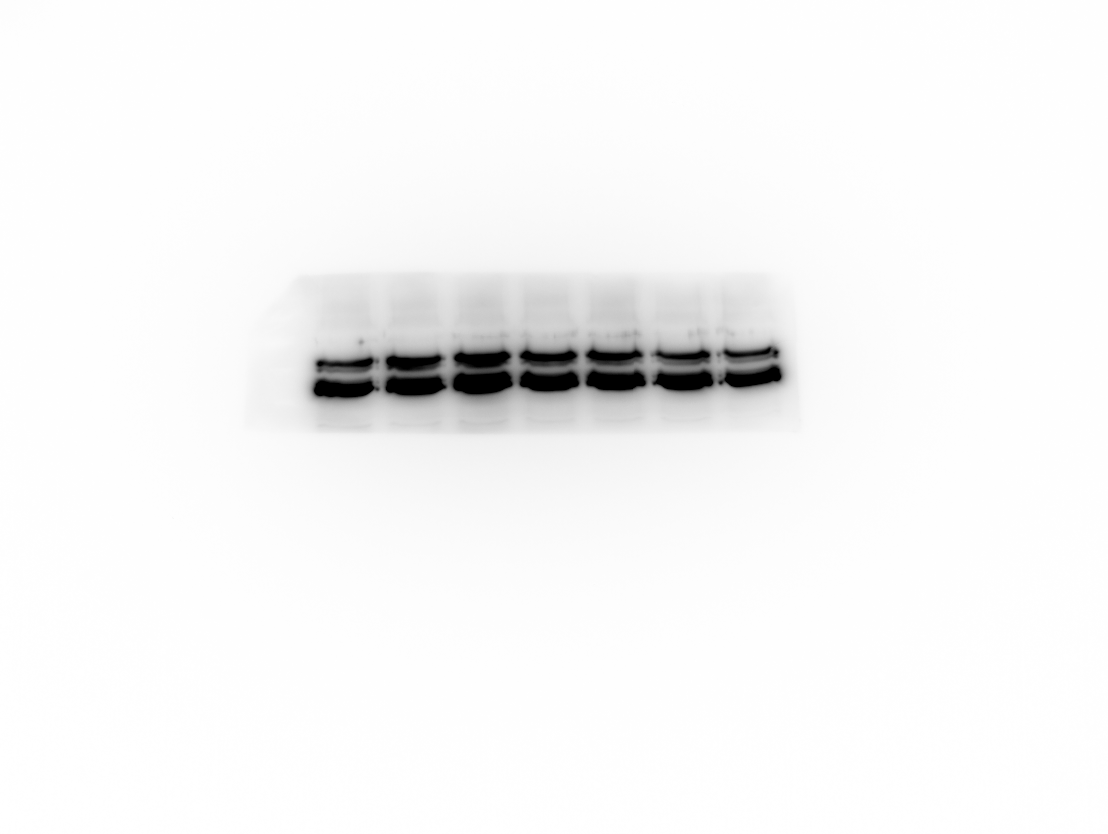

Supplement: Supplementary file 2 [file DataSheet_2.zip › supplementary material Figure 6-JNK.tif]

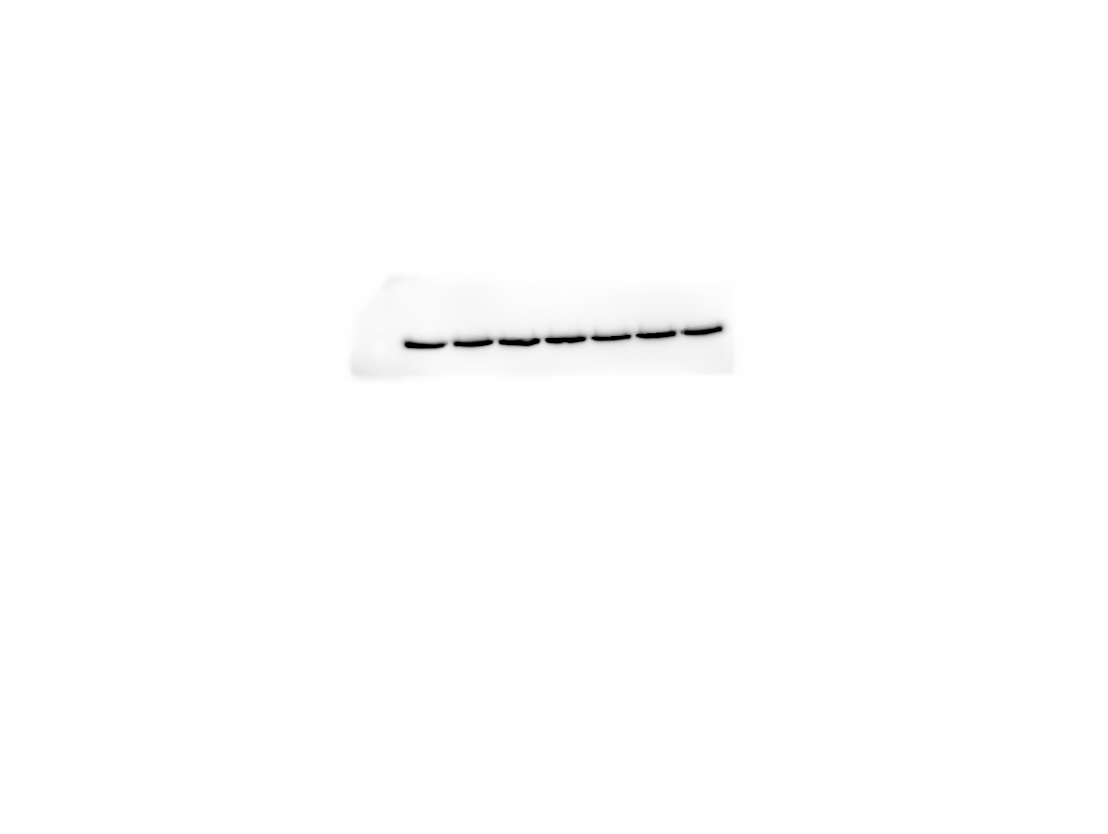

Supplement: Supplementary file 2 [file DataSheet_2.zip › supplementary material Figure 6-p38.tif]

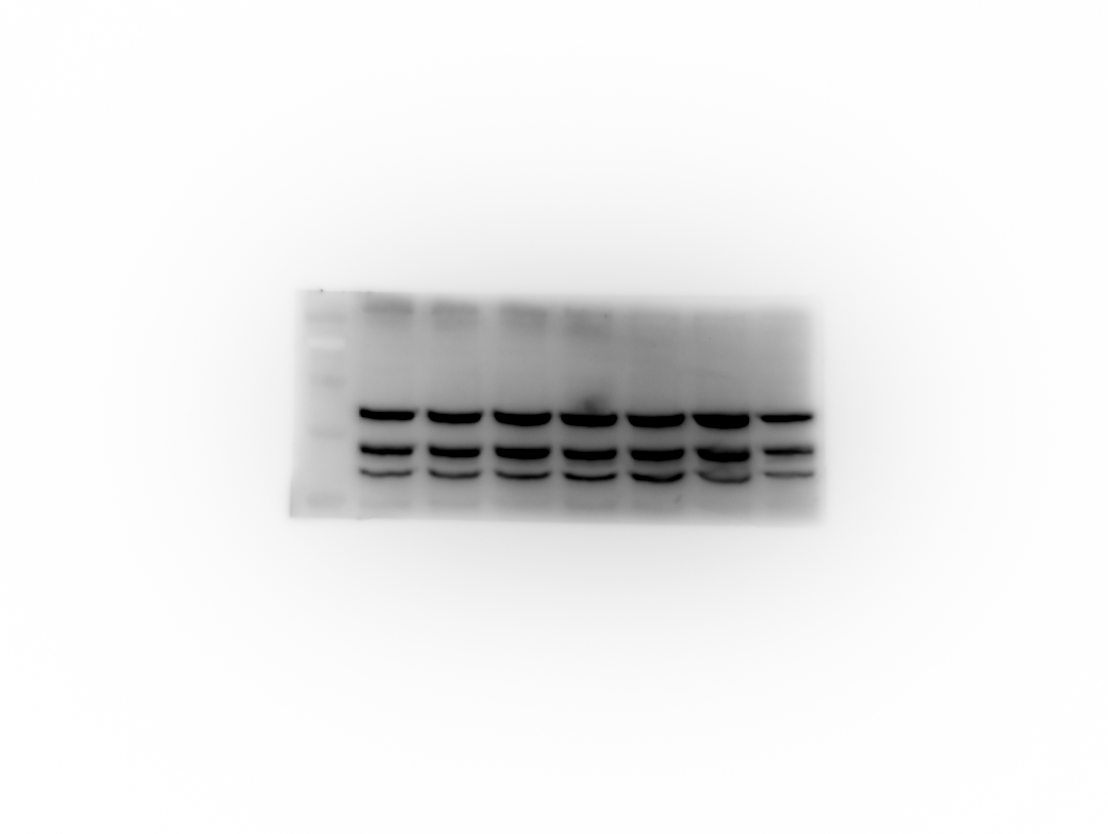

Supplement: Supplementary file 2 [file DataSheet_2.zip › supplementary material Figure 6-pERK.tif]

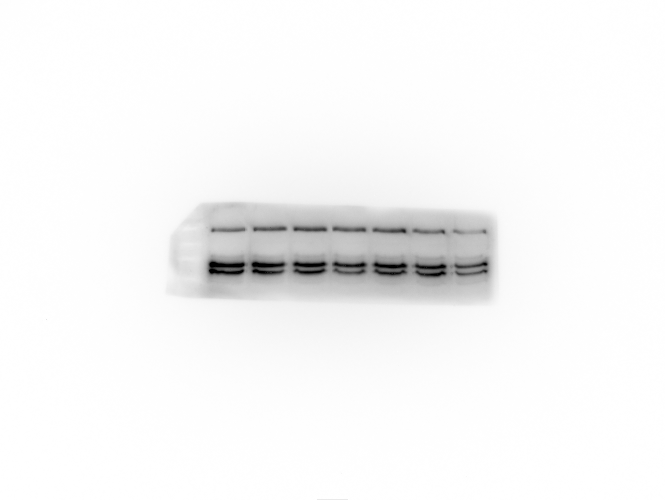

Supplement: Supplementary file 2 [file DataSheet_2.zip › supplementary material Figure 6-pJNK.tif]

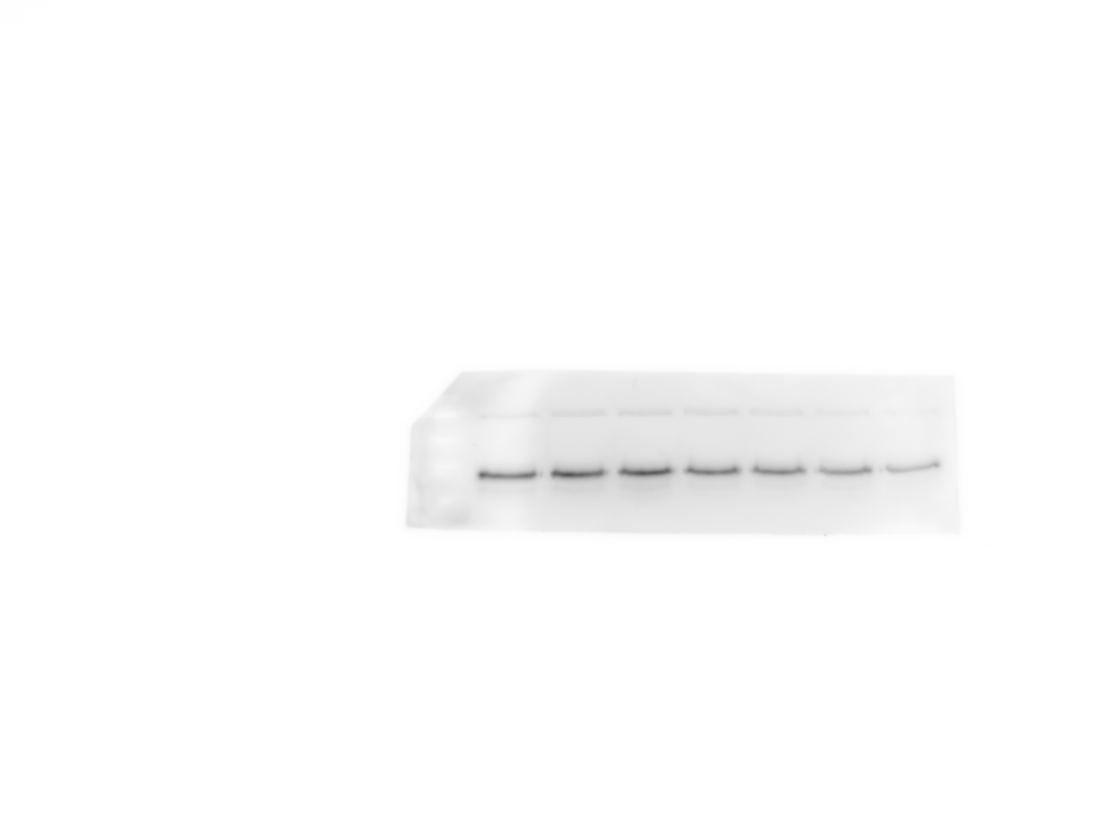

Supplement: Supplementary file 2 [file DataSheet_2.zip › supplementary material Figure 6-pp38.tif]

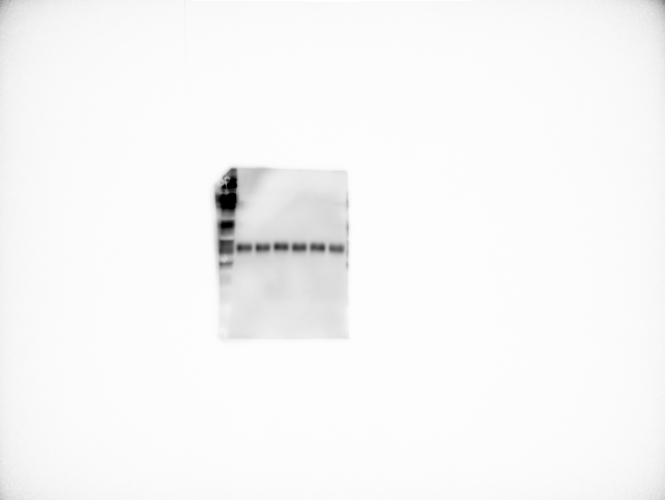

Supplement: Supplementary file 2 [file DataSheet_2.zip › supplementary material Figure 2-GAPDH.tif]

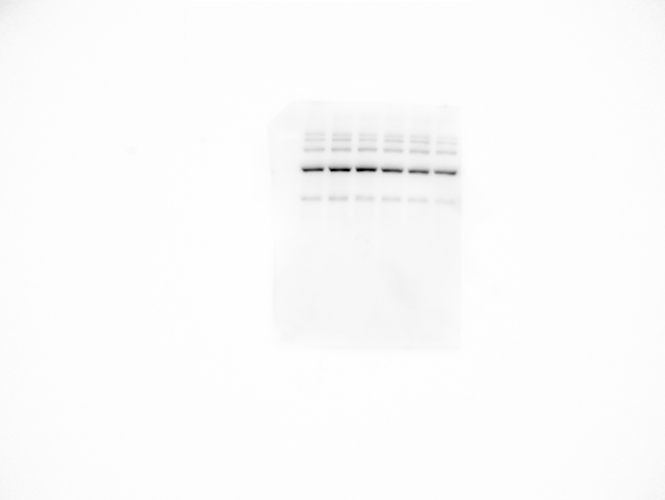

Supplement: Supplementary file 2 [file DataSheet_2.zip › supplementary material Figure 2-MITF.tif]

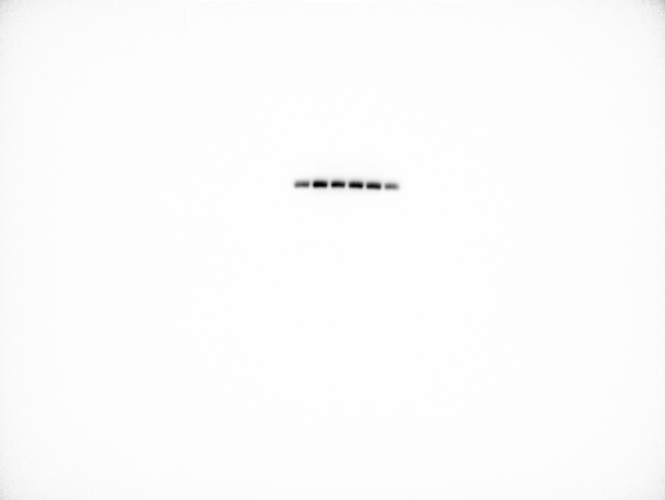

Supplement: Supplementary file 2 [file DataSheet_2.zip › supplementary material Figure 2-TRP-1.tif]

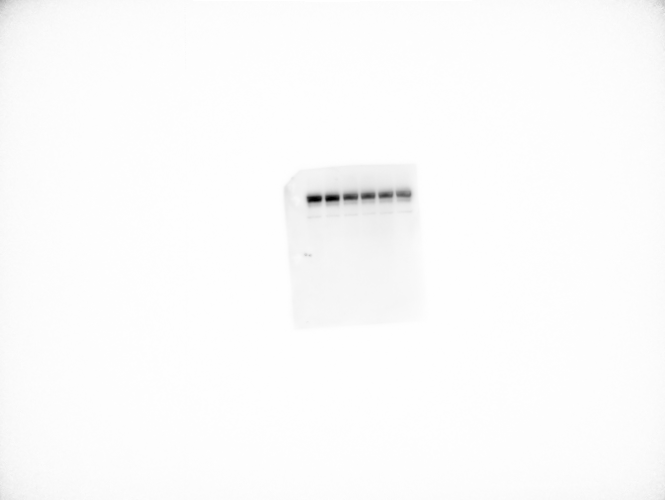

Supplement: Supplementary file 2 [file DataSheet_2.zip › supplementary material Figure 2-TRP-2.tif]

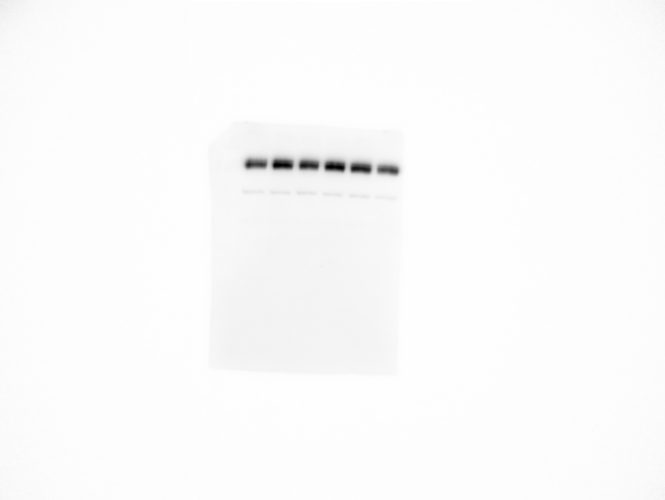

Supplement: Supplementary file 2 [file DataSheet_2.zip › supplementary material Figure 2-Tyrosinase.tif]

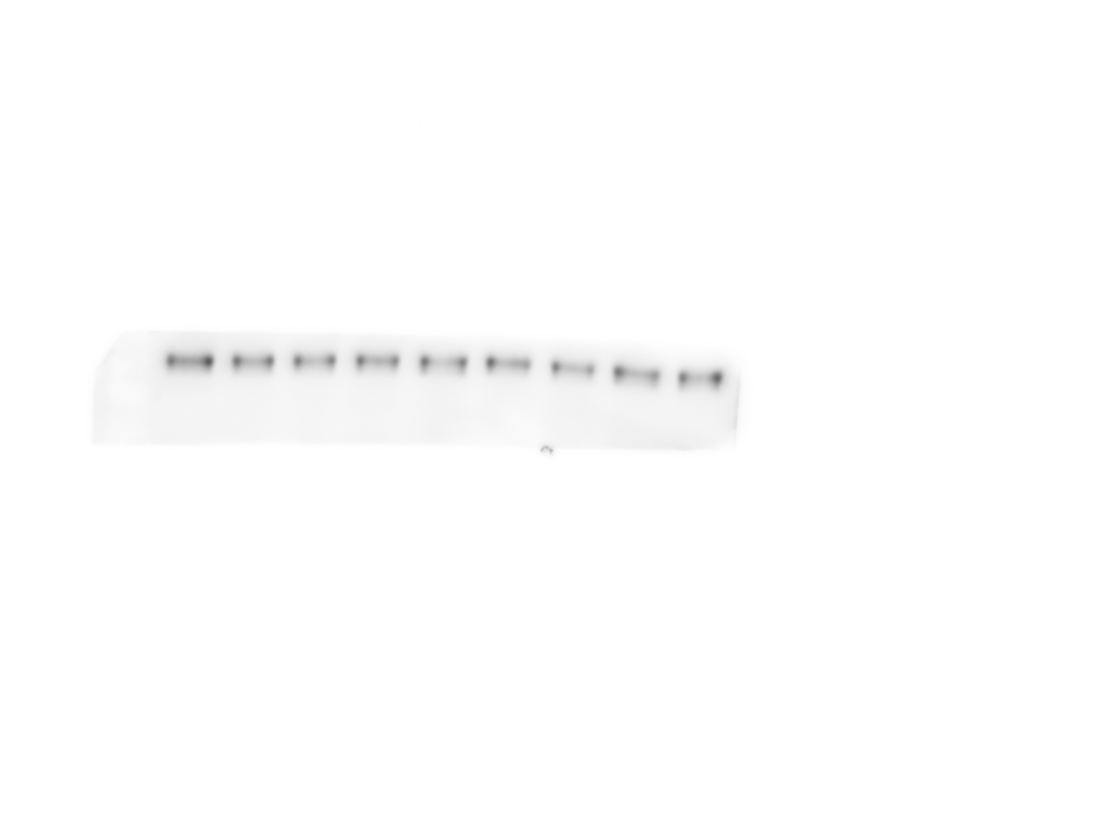

Supplement: Supplementary file 2 [file DataSheet_2.zip › supplementary material Figure 4(D)-GAPDH.tif]

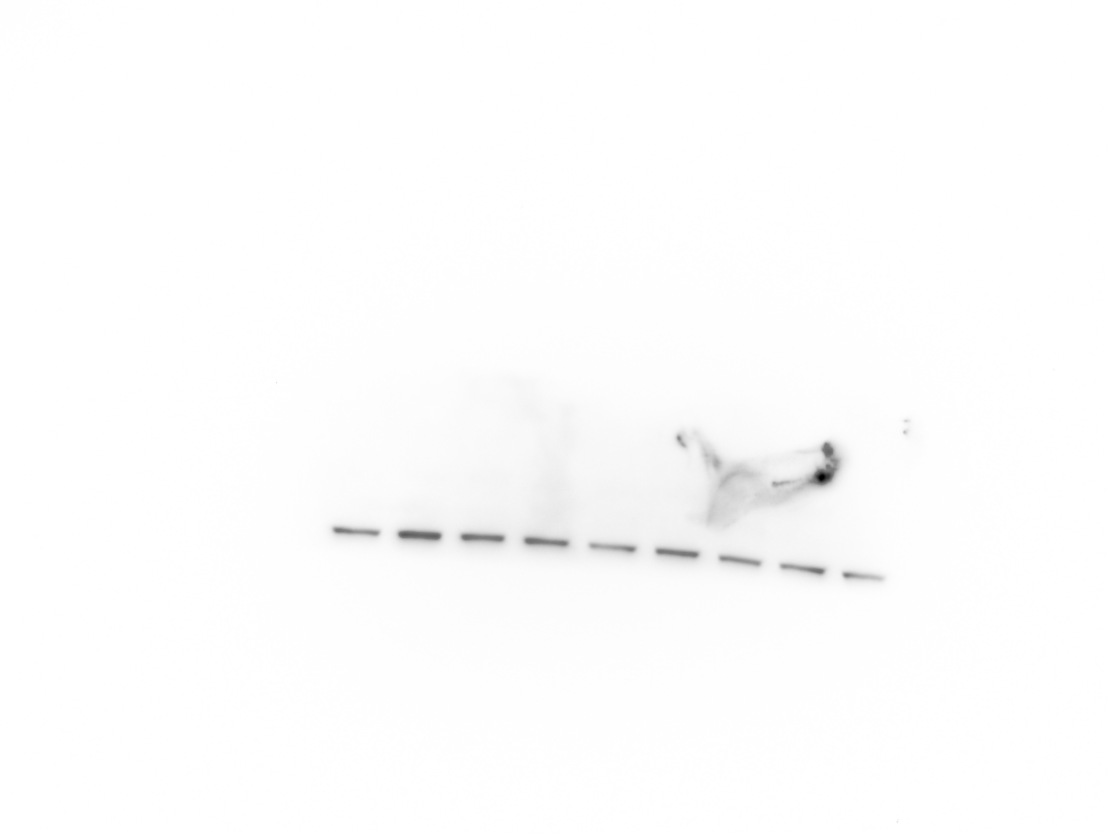

Supplement: Supplementary file 2 [file DataSheet_2.zip › supplementary material Figure 4(D)-MITF.tif]

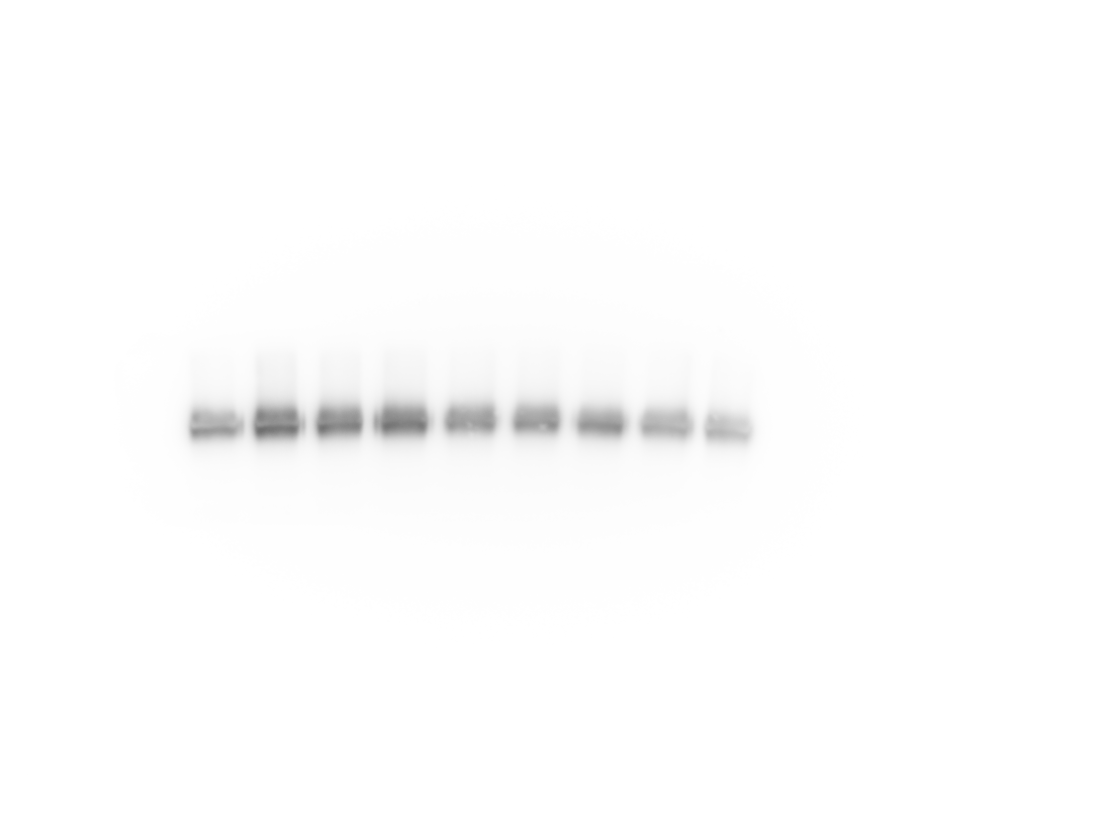

Supplement: Supplementary file 2 [file DataSheet_2.zip › supplementary material Figure 4(D)-TRP-1.tif]

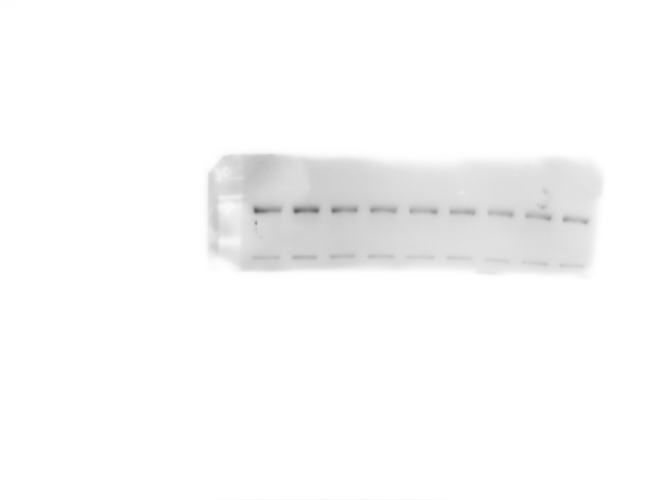

Supplement: Supplementary file 2 [file DataSheet_2.zip › supplementary material Figure 4(D)-tyrosinase.tif]

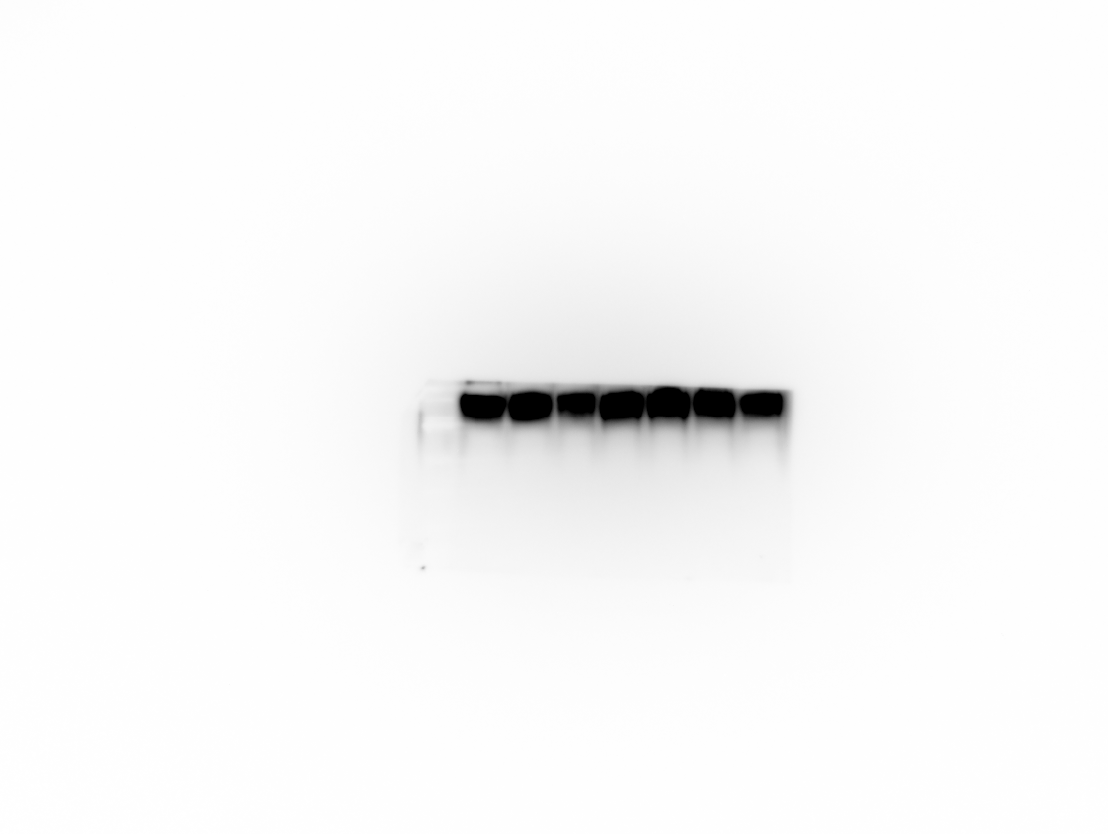

Supplement: Supplementary file 2 [file DataSheet_2.zip › supplementary material Figure 5(A)-CREB.tif]

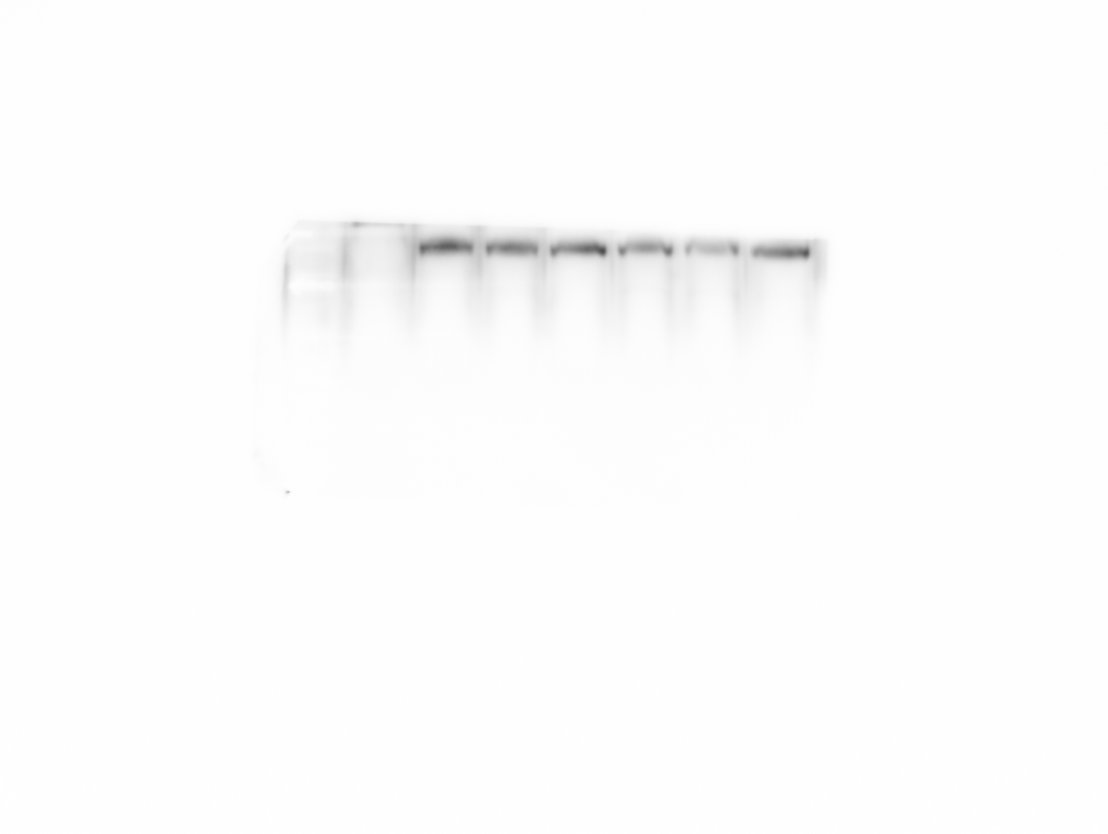

Supplement: Supplementary file 2 [file DataSheet_2.zip › supplementary material Figure 5(A)-pCREB.tif]

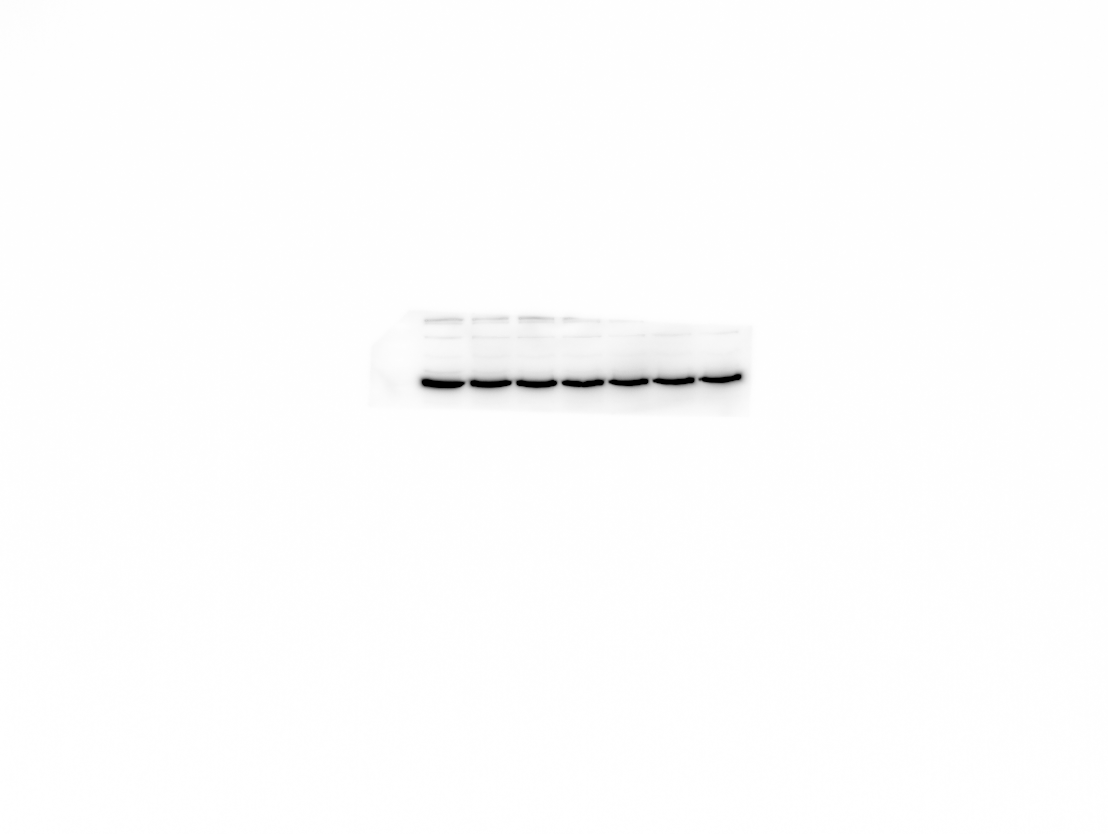

Supplement: Supplementary file 2 [file DataSheet_2.zip › supplementary material Figure 5(A)-PKA.tif]

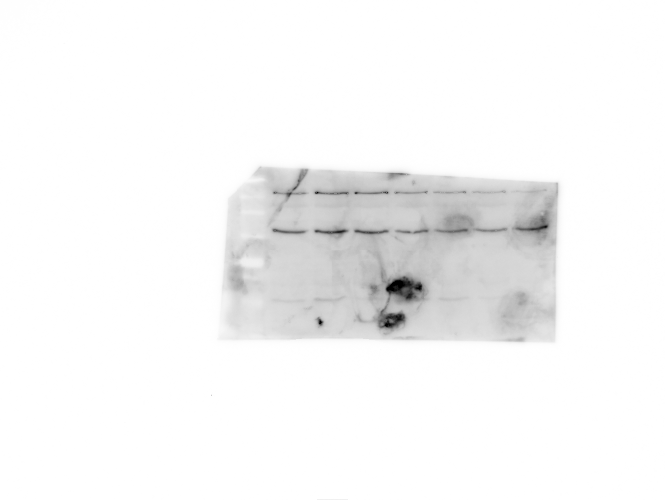

Supplement: Supplementary file 2 [file DataSheet_2.zip › supplementary material Figure 5(A)-p-PKA.tif]

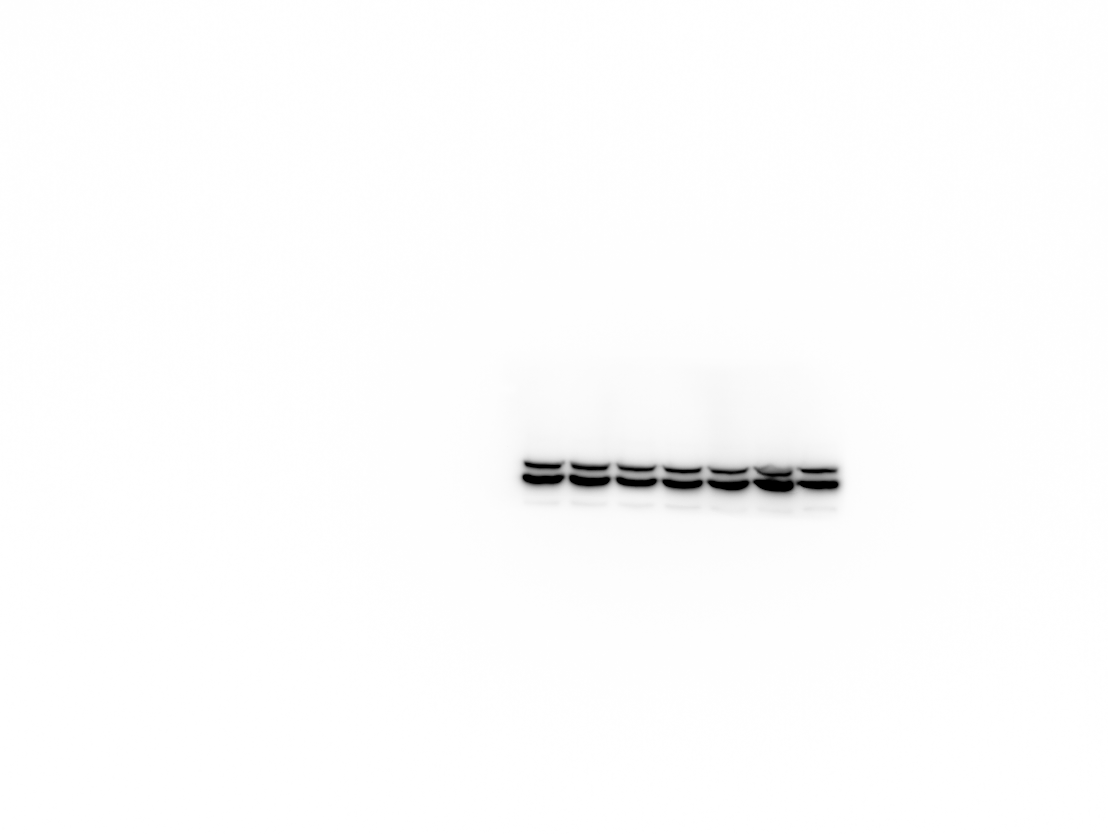

Supplement: Supplementary file 2 [file DataSheet_2.zip › supplementary material Figure 6-ERK.tif]
